# Planning and licensing for marine aquaculture

**DOI:** 10.1111/raq.12783

**Published:** 2023-01-11

**Authors:** Lynne Falconer, Karl Cutajar, Amalia Krupandan, Elisa Capuzzo, Richard A. Corner, Tim Ellis, Keith Jeffery, Eirik Mikkelsen, Heather Moore, Francis X. O'Beirn, Pauline O'Donohoe, Neil M. Ruane, Robyn Shilland, Paul Tett, Trevor C. Telfer

**Affiliations:** ^1^ Institute of Aquaculture, University of Stirling Stirling Scotland UK; ^2^ Centre for Environment, Fisheries and Aquaculture Science Dorset UK; ^3^ School of Ocean Science, Bangor University Wales UK; ^4^ Nofima Tromsø Norway; ^5^ Agri‐Food and Biosciences Institute Belfast UK; ^6^ Marine Institute, Oranmore, Co. Galway Ireland; ^7^ The Association for Coastal Ecosystem Services Lochend Cottage Dunbar UK; ^8^ Scottish Association for Marine Science Oban UK

**Keywords:** aquaculture planning, environmental management, licensing, regulation, sustainable development

## Abstract

Marine aquaculture has the potential to increase its contribution to the global food system and provide valuable ecosystem services, but appropriate planning, licensing and regulation systems must be in place to enable sustainable development. At present, approaches vary considerably throughout the world, and several national and regional investigations have highlighted the need for reforms if marine aquaculture is to fulfil its potential. This article aims to map and evaluate the challenges of planning and licensing for growth of sustainable marine aquaculture. Despite the range of species, production systems and circumstances, this study found a number of common themes in the literature; complicated and fragmented approaches to planning and licensing, property rights and the licence to operate, competition for space and marine spatial planning, emerging species and diversifying marine aquaculture production (seaweed production, Integrated Multi‐Trophic Aquaculture [IMTA], nutrient and carbon offsetting with aquaculture, offshore aquaculture and co‐location and multiuse platforms), and the need to address knowledge gaps and use of decision‐support tools. Planning and licensing can be highly complicated, so the UK is used as a case study to show more detailed examples that highlight the range of challenges and uncertainty that industry, regulators and policymakers face across interacting jurisdictions. There are many complexities, but this study shows that many countries have undergone, or are undergoing, similar challenges, suggesting that lessons can be learned by sharing knowledge and experiences, even across different species and production systems, rather than having a more insular focus.

## 1 INTRODUCTION

Marine aquaculture, also known as mariculture, is suggested as a route to substantially increase food production in coming decades.^1^, ^2^ High‐level global policies are needed to promote marine aquaculture within the food production system, but as marine aquaculture farms must comply with the regulatory requirements of the jurisdiction in which they are located, the sector's ability to increase food production is also conditional on decisions within more local‐level planning and licensing systems.

As an initial part of the regulatory process, many countries or jurisdictions set location and production limits by granting a licence or licences. Aquaculture production cannot take place without these licences, as they govern the level of production, the degree of environmental impact acceptable, and the location of the farm. These parameters are often decided through initial environmental impact assessments,^3^ environmental capacity modelling, local acceptability evaluation, and sometimes integration within the wider local and regional marine planning process where these exist. After development and commencement of production, farms are often subject to environmental monitoring to ensure that the conditions of the licence are adhered to. If not, action or mitigation measures must be taken to keep the licence. This forms part of the aquaculture regulatory process.

Marine aquaculture covers a diverse range of species, each with different biological requirements and environmental interactions, as well as an array of production technologies and farm management strategies, which also give rise to different economic and social impacts. For any type of aquaculture, a suitable site is a prerequisite for a successful business and is fundamental to achieving sustainability.^4^ However, finding a suitable location is complicated given the range of requirements and a need to balance environmental sustainability, carrying capacity, health and welfare of the farmed species, economic production potential of an operation, and societal approval. Another challenge for marine aquaculture development is that planning policies and regulations vary considerably between jurisdictions, with no uniform approach existing for aquaculture planning. The variation is both in the approaches and systems for planning and regulation, and in the stringency of regulations. The uneven approaches to licensing and regulation have contributed to regional differences in aquaculture growth and expansion throughout the world.^5^, ^6^ Planning, licensing, and regulation have been identified as major bottlenecks for future development.^7^ Good governance is also essential for social acceptance of marine aquaculture,^8^ but aquaculture licensing has become a contentious regulatory and social issue in some countries. Clearly, given the complexities of aquaculture, establishing a robust and fair licensing system with supporting regulation is a challenge, but is needed. A ‘one‐size‐fits‐all’ approach will either have too much or too little control, will not relate to differences in values amongst societies, and may not encourage sustainable development.^9^ Since there are differences in management and necessary control depending on species grown, some level of differentiation is important, even in the context of overarching regulations.

The need to review, revise and in some cases, establish new planning and regulatory systems for marine aquaculture is recognised throughout the sector. In May 2021, The European Commission^10^ published their ‘*Strategic guidelines for a more sustainable and competitive EU aquaculture for the period 2021 to 2030*’. The need to improve licensing and regulatory frameworks is a key part of that vision, although such improvement has been an overarching theme within the European Parliament since at least 2009.^11^ Recent national reviews in countries such as Scotland,^12^, ^13^ England^14^ and Ireland,^15^ have highlighted the need to update and streamline the licensing processes. Similarly, industry press has reported calls for reform from finfish and shellfish producers,^16^, ^17^ and commitments from politicians and governments for further reviews and restructuring.^18^, ^19^ Political will and political agenda play a major part in aquaculture policy and regulation.^20^ Regulatory reviews often take place in complicated political landscapes, especially when there are changes in governing political party or new policy directions. In the case of the United Kingdom of Great Britain and Northern Ireland (UK), reviews of policy and regulation are affected by ‘Brexit’, and many of the consequences of leaving the European Union (EU) are still unclear and may take several years to resolve.^21^, ^22^ Lengthy reviews and inertia can affect the sustainable use of marine resources, while reviews that are conducted too quickly are unlikely to have the level of detail needed to identify suitable reforms. If countries are looking to revise regulations, then it is important to consider the issues and approaches that other jurisdictions have as a learning mechanism for potential routes to overcome difficulties and strengthen their regulation and licensing systems.

This review aims to evaluate the challenges of planning and licensing for growth in sustainable marine aquaculture. First, a broad assessment of recent scientific literature is used to identify some of the key issues associated with marine aquaculture planning, licensing and regulation frameworks throughout the world. Next, the UK is used as a case study, including examples of recent policy documents, to demonstrate the challenges encountered by industry, regulators and other decision makers in developing a sustainable marine aquaculture sector.

## 2 REVIEW OF RESEARCH ARTICLES AND ACADEMIC STUDIES

A literature search was performed to gain an understanding of the issues raised in academic studies concerning marine aquaculture planning and licensing. The initial search followed a structured approach, but aimed to find relevant literature for the topic rather than perform a strict quantitative study or meta‐analysis. The search of the Scopus database in May 2021 used the terms ‘*aquaculture*’ AND ‘regulat*’ OR ‘licen*’ OR ‘*planning*’ OR ‘*governanc*e’ OR ‘*policy*’ OR ‘*environment* AND *sustainability*’, aimed at the article title, abstract and keywords. This initial search was deliberately wide to capture as many relevant studies as possible. The search results were imported into the Rayyan web app^23^ for initial screening of titles and removal of duplicate studies. The next step involved reading the abstracts to identify potentially relevant scientific articles, and then full texts to determine if they were relevant in the scope of this study. Additional studies and grey literature were identified from literature cited within those texts, as well as others known to the authors that may not have been found within the original search limits.

Since licensing and regulation can change over time, research articles published since 2010 were of primary focus to provide a more state‐of‐the‐art overview, although other relevant documents were included when they provided important context. The scope of the topic is vast, but there were some limitations as some topics/subject areas may be more popular than others at any given time. Considering the region's long history of mariculture production, the search returned proportionally fewer studies from Asia than originally expected. Some relevant articles from Asia may have been missed due to the timeframe of the search, and limiting the search to studies in the English language. This review focuses on planning and licensing approaches for marine aquaculture, but it is important to recognise that there are other regulatory considerations not considered that will also affect the development of aquaculture; bivalve transfers,^24^ fish welfare^25^ and use of veterinary medicines^26^ are some examples.

Some common themes were found in the literature; related to complicated and fragmented approaches to planning and licensing, property rights and the licence to operate, competition for space and marine spatial planning, emerging species and diversifying marine aquaculture production (e.g., seaweed production, Integrated Multi‐Trophic Aquaculture [IMTA], nutrient and carbon offsetting with aquaculture, offshore aquaculture, co‐location and multiuse platforms), and the need to address knowledge gaps and use of decision support tools. The following sub‐sections provide an overview of these themes. The main findings (both challenges and opportunities) within these themes are summarised in Table 1.

**TABLE 1:** Summary of themes and findings from a literature review of the challenges and opportunities within planning and licensing for growth of sustainable marine aquaculture throughout the world

Theme	Main findings (challenges and opportunities)
Complicated and fragmented approaches to planning and licensing	Lack of coordination between relevant agencies
	Lack of comprehensive, available information about licensing frameworks and processes for applicants
	Low willingness within and among governments to address issues
	Variation between areas applying same laws and frameworks
	Diverse nature of aquaculture industry as a barrier to streamlining licensing systems
	Legislation not always fit for purpose; aquaculture‐specific legislation not always present
	Lack of regional (multinational) policy coherence
	Conflicting agendas of various government agencies involved
	Opportunity to use planning and licensing to drive social change and technological innovation
Property rights and licence to operate	The marine environment as a ‘common pool resource’ creates challenges for rights to operate
	Multiple stakeholder interests and rights to be balanced, including indigenous rights
	Prescriptive and inflexible licences make adaptation to changing environments challenging
	Appeals process can be both beneficial to applicant or can raise challenges in balancing stakeholder views
	Financial value of use of space may be assessed and ‘taxed’, which can be complex
	Variability in licence term between jurisdictions
	Evolving legislation and its impact on existing operations
	Disconnect between timeframes and requirements of licensing, and those of necessary finance and investment cycles
	Unlicensed/illegal operations
Competition for space and Marine Spatial Planning (MSP)	Time and resource requirements (of governments and planning and regulatory bodies) of MSP
	Knowledge gaps and uncertainties hinder development of marine spatial plans
	Static nature of MSPs make them unresponsive to changing environments
	Potential for Allocated Zones for Aquaculture (AZAs) within MSPs
	Potential for use of tools and models to aid MSP and identify suitable AZAs
	Potential use of AZAs for regional‐scale management measures, but risk of unfair penalties because of the actions of others within the region
	Potential for local‐scale planning for gaining local acceptance
Emerging species and diversifying marine aquaculture production	
Seaweed farming	Potential for ecosystems services to help take up nutrient and carbon and mitigate greenhouse gases
	Underdeveloped outside Asia
	Lack of appropriate licensing and regulation frameworks (requirement for sufficient space in inshore waters)
	Knowledge and data gaps
Integrated Multi‐Trophic Aquaculture (IMTA)	Multiple potential benefits for business and environment
	Dynamic nature of marine environment makes planning of IMTA challenging
	Insufficient licensing and regulatory systems, hindering development of sector
	Potential for regional (rather than site‐based) IMTA systems
	Need for proof‐of‐concept to stimulate investment
Offsetting with aquaculture	Potential for extractive species to remove excess nutrients
	Potential for carbon offsetting using aquaculture, particularly seaweed
	Financial challenges if operations solely financed by offsetting
	Scientific and technical challenges of certified crediting schemes; underdeveloped field for marine environments and aquaculture in particular
	Benefits of aquaculture to delivery of ecosystem services not always recognised and accepted by communities
Offshore aquaculture	Lack of strong planning framework; existing regulations focus on inshore rather than offshore developments
	Lack of capacity and resources amongst agencies to adapt to changing context
	Uncertainty in licence assessments leads to precautionary rejection of applications
Co‐location and multi‐use platforms	Complex to assess costs and benefits; all stakeholders need clear benefits to commit
	Potential to utilise decommissioned oil and gas platforms
	Cumulative and interactive impacts and needs difficult to assess and monitor
The need to address knowledge gaps and use of decision support tools	Planning and licensing can obstruct or facilitate innovation
	Potential for development licences to evaluate high‐cost and high‐risk developments
	Variable drive to adopt use of decision support tools
	Available decision support tools largely focus on salmon aquaculture
	High cost and complex requirements of decision support tools hinders their uptake
	Often a lack of adequate data to feed into tools
	Potential for use of Earth Observation data, but scientific and technical challenges remain
	Potential for in situ data collection to fill data gaps

### 2.1 Complicated and fragmented approaches to planning and licensing

One of the most frequent themes in recent literature is that planning and licensing processes are often complicated and fragmented, with multiple licences required through multiple agencies which are not well coordinated. This does not seem to be an issue unique to a particular species or jurisdiction. Studies citing complicated or disjointed approaches to planning and licensing included (but were not limited to): finfish in Ireland, New Zealand, Norway and Scotland,^27^, ^28^, ^29^, ^30^ and shellfish in Brazil, Ireland and South Africa.^31^, ^32^, ^33^, ^34^ In an extensive stakeholder consultation study across 16 locations throughout the world, Galparsoro et al.^7^ found that administrative procedures/licensing, planning and management, and regulation were all amongst the most‐frequently cited issues preventing expansion of the sector.

The complicated nature and the costs of planning and licensing systems have also been highlighted as barriers for new entrants and small‐scale farmers. Scottish scallop fishermen who were seeking to move into aquaculture may have given up due to regulatory challenges.^35^ Small‐scale mussel and oyster farmers in South Africa struggled in a regulatory environment that was more suited to larger‐scale enterprises.^31^ While overly complex regulations and long administrative processes in Brazil favoured companies with more resources and experience, therefore reducing opportunities for individuals and smaller companies.^34^

In many countries, agencies and authorities do not engage in coordinated planning, but make decisions based on departmental responsibilities. This is further complicated in countries (e.g., Italy, Israel, Cyprus) where the agencies involved are not specifically set up for aquaculture, but for wider, overarching, purposes (e.g., food production) and thus have a broader range of competencies and experiences, but lack the necessary competence on aquaculture.^36^ In a review of aquaculture development in the Near East and North Africa, FAO^37^ noted that conflict of interest can occur among the different authorities involved in governance and regulation, which may lead to poor management, strategies and policies. This conflict has been seen in Norway, between coastal zone and aquaculture management.^38^, ^39^ Lack of effective inter‐institutional mechanisms at local, national and regional levels can hinder effective communication, and results in lengthy decision‐making processes for aquaculture licensing.^40^

Information about legislative frameworks is often scattered and difficult to find, available in different formats and frequently accessible only in the relevant national language. On occasion, it is not publicly accessible at all. At the regional level, information on legislation and marine resources can be compiled in databases and repositories (e.g., General Fisheries Commission for the Mediterranean Regional Repository [GFCM‐LEX]) to facilitate accessibility, transparency and reliability. If regulations are complicated and lack clarity, aquaculture producers must spend more time and financial resources identifying what rules apply and how to comply with them.^41^ In a survey of shellfish farmers on the Pacific coast of the United States,^42^ respondents knew of farms that had gone out of business as a result of administrative and operational burden because of the confused regulatory landscape. Challenges are also seen in the Mediterranean and Black seas, where it is recognised that aquaculture development is constrained by lack of clarity in regulatory and licensing requirements, that then lowers investment potential.^40^ In addition to cost and time implications, complicated and confusing regulations undermine confidence in the system and can lead to a lack of trust from stakeholders and the public.^43^ Hishamunda et al.^44^ noted that globally, as with many forms of governance, corruption is more prevalent where there is a lack of transparency.

In many areas, there is a perception from aquaculture stakeholders that national governments are not investing sufficient time or effort to address issues with licensing and regulatory frameworks.^7^ Within the European Union, issues of licensing, regulation and the time needed to gain permissions have been recognised since at least 2009.^45^ One of the problems is the number of different agencies involved and a lack of clarity over their respective responsibilities. Osmundsen et al.^30^ and Stokke^39^ noted that the number of different agencies involved in regulating Norwegian salmon aquaculture can lead to confusion and regulatory competition over some issues. Carr^27^ reported that there was a view amongst Irish stakeholders that poor communication and narrowly defined remits of different regulatory agencies led to lack of oversight in the salmon sector. The stakeholders suggested that there was a need for reform, with a single organisation taking a larger role to develop and implement regulations more holistically.^27^ However, Norwegian salmon stakeholders interviewed by Osmundsen et al.^30^ said there still can be inconsistencies in how decisions are made by a single state agency across different regional offices.

In some locations, there are conflicting remits within the same organisation. For example, an organisation may be responsible for environmental policy and protection, while at the same time being responsible for approval of activities, including aquaculture, that may have some detrimental effects on the environment, even if those effects are not permanent. In other cases, committees with representatives from research institutions, aquaculture industry and administrations at local and regional level have been established to improve institutional support and coordination, for example, in Andalusia in Spain.^46^ At a regional level, the General Fisheries Commission for the Mediterranean (GFCM) has identified a lack of basic comparable regulations for licensing and certification. Regional Fisheries Management Organisations (RFMOs) can harmonise specific practices and improve coherence.^40^

A one‐stop‐shop for licence applications at the national level is often recommended to streamline the process.^44^ This could be established in a range of different formats, but the key aim should be to support an efficient and clear licensing process, accounting for differences in likely impacts and species. Hishamunda et al.^44^ noted that a one‐stop‐shop does not have to involve full institutional integration, but can be an access point for guidance and advice, with all information located in one place.

Evidently, it would be in everyone's interest to have more straightforward, streamlined and transparent licensing systems. However, aquaculture is a complex industry that spans many areas that are subject to regulation; consequently there are a multitude of different statutory procedures and stakeholder interests that need to be balanced to ensure effective overall governance. Osmundsen et al.^30^ outlined how the ‘wicked’ nature of aquaculture means that regulation and management of the sector is a challenge. The term ‘wicked problem’ refers to complicated issues that may have many different factors and uncertainties that make it difficult to both define and find a solution.^47^, ^48^ Indeed, there may be no objectively ‘right’ solution, and both problem definition and problem solution may be contested by stakeholders.

For aquaculture planning and licensing, the fundamental questions are: where can aquaculture sites be developed (spatial planning, zoning and site selection), and how much production can occur? At the heart of these questions is the concept of carrying capacity,^4^ which can be categorised into at least four different pillars: physical, ecological, production and social. In most cases, decisions will have to be made that involve trade‐offs and compromises across the different pillars.^49^ Good governance, at the national and regional levels, requires the relevant authorities to define and communicate expectations and limitations, and provide appropriate mechanisms and tools to support sustainable development.^44^

Some countries have aquaculture‐specific legislation, while in others, aquaculture is governed under broader laws (e.g., environmental management) or those originally developed for fisheries and/or agriculture. For example, Young et al.^50^ highlighted that Norway, Iceland and the Faroe Islands have aquaculture‐specific legislation, but Canada and Sweden do not. One of the drivers for countries having specific aquaculture legislation is the importance of the sector within the jurisdiction, as development and implementation of aquaculture‐specific legislation can be complex and expensive.^44^ In Canada, there is recognition from industry and regulators that aquaculture and wider Blue Growth ambitions would benefit from an aquaculture‐specific Act, and work is underway to develop one.^51^ Likewise, the GFCM has advocated for specific laws on aquaculture to improve existing regulatory and administrative frameworks.^52^ Specific provisions that have been proposed include coordinated planning and the establishment of Allocated Zones for Aquaculture (AZA).

The aquaculture licensing framework often transcends different spatial scales, with national and local legislation playing a role.^30^ While diversity in policies and strategies is expected at a national level to meet an individual country's sectoral needs, policy coherence has recently been suggested as a means for promoting aquaculture development, particularly at a regional level (e.g., Western Indian Ocean—see Brugere et al.^53^). However, it is important to note that regulatory barriers at a regional, federal or multinational level (e.g., EU) would be more complex than at a national level, due to the lengthy, comprehensive processes required for amendments to legislation. The GFCM in the Mediterranean and Black seas has adopted Resolution GFCM/41/2017/2 on guidelines for streamlined authorization and leasing processes for aquaculture development. This provides advisory guidelines and minimum common criteria. They aim to propose common definitions, standards and reference documents and promote soft‐law mechanisms to simplify administrative procedures.^54^

Regardless of the legal mechanisms, relevant authorities must aim to find a way to promote socio‐economic benefits while minimising negative environmental impacts. If licensing and regulation are too restrictive, then development of the aquaculture sector can be affected; as shown by Abate et al.^5^ Furthermore, Abate et al.^55^ showed that growth of the industry is influenced by relative decision‐making power amongst the various regulatory agencies involved. For instance, aquaculture production increased in Vietnam, Myanmar and China, where more decision‐making power is available to food production agencies, but hindered in countries and regions where environmental agencies have more regulatory power and different priorities than food production agencies (e.g., The United States, Japan and EU). Mäkinen et al.^56^ noted that the implementation of strict environmental regulation in Finland led to companies moving their businesses and investments to other countries.

It is not a surprise that businesses may choose to move elsewhere, especially large multinational companies that operate across countries, as in the salmon industry. Business scale is then an issue, since smaller companies or individuals do not have the same resources available, so their options to react to regulatory demands will be more limited. Chávez et al.^9^ suggested that the regulatory design process should consider the structure of the industry, how domestic and multinational companies may respond to conditions (e.g., increase, decrease or move production), and the subsequent implications on socio‐economics and other factors such as disease risks. Aanesen and Mikkelsen^57^ showed how local ownership of aquaculture companies operating in a region may be crucial to ensure that the local net benefits from aquaculture are positive when both environmental impacts and economic benefits are considered. This may also be important for the local social acceptance of aquaculture.

Planning and licensing have been used as mechanisms to improve or advance specific aspects of the sector. The allocation process of commercial salmon aquaculture licences in Norway has had different stated priorities over the years,^58^ affecting different groups ranging from fishermen and farmers in coastal regions with employment problems, small aquaculture companies, fish farmers in specific counties or municipalities, to support of indigenous people and those who promise to reduce fish disease problems. Each group will have their own approach to aquaculture and different ways of operating that can enhance or limit innovation. Innovation can take different forms, have a range of complexity and involve organisations within and external to the aquaculture sector.^59^ Nonetheless, it is important to note that the capacity for innovation will depend on the capacity of different companies to innovate, particularly for less‐profitable sectors such as shellfish and for emerging seaweed producers. Greaker et al.^60^ suggested that stricter regulations could, in some cases, promote innovation through the development of new and improved techniques that assist with compliance. Conversely, Engle and Stone^41^ suggested that innovation can be suppressed by strict regulation as companies spend time and resources trying to conform to regulatory demands rather than considering other available options. The study by Greaker et al.^60^ focused on innovation in the Norwegian salmon sector, particularly regarding control of sea lice, and noted that government support had been essential for some innovations, but the lack of support for others meant they were unlikely to reach commercial‐scale application. In some countries, licensing and regulatory systems have mechanisms that can facilitate innovation; for example, some of the Norwegian special purpose licences encourage innovative development,^61^, ^62^ which is discussed more in Section 2.5. For EU aquaculture, Guillen et al.^63^ noted that a move from the more commonly used ‘command and control’ regulation (where standards and limits are set) to more incentive‐based regulation (e.g., taxes on environmental impact under the Polluter Pays Principle) might be a potential way of overcoming the lack of growth and encouraging innovation and better practices.

### 2.2 Property rights and the licence to operate

Property rights within marine environments are a complex issue, and are a commonly occurring theme within the aquaculture scientific literature.^64^ Though some areas may be under private ownership, in most cases marine environments are a common good which belongs to everyone. Thus, property rights refer to the space in which marine aquaculture activities take place.^64^ Understandably, property rights can be contentious, as different stakeholders will have a range of views on how the natural environment can and should be used, as well as who has the rights to that use. A particularly complex issue is the rights of Indigenous populations^50^ including many First Nations groups in Canada, and Sami people in Norway, who depend on coastal areas for livelihoods, and for whom the coast and sea can have cultural and spiritual significance.^65^, ^66^, ^67^, ^68^ Young et al.^50^ discuss how representation varies depending on location, about uncertainty on rights and how Indigenous people are, or are not, involved in aquaculture development decisions. Such contentions fit within an overall narrative of issues regarding access and rights to coastal areas for Indigenous peoples.^69^

In most countries, aquaculture producers obtain licences from relevant authorities which grant permission for development and use of a site. Licences are important as a mechanism to achieve sustainable development within an area, and they strengthen the position of the aquaculture producer. As pointed out by Renwick,^70^ unlike in agriculture where the farmer owns the land, in marine aquaculture the licence to farm is the only security that operators have in most coastal and marine environments. The licence to operate, or the production licence (which may also be known as a permit) will typically outline the conditions to which the producer must comply to use the space for production. How specific and strict each licence is, will depend on the governing authority and legal framework.^44^ In previous decades in China, many licences did not specify species, stocking densities or system layouts, which led to unrestricted development and eventually resulted in environmental and disease challenges.^71^ Licences that have more detailed conditions can provide more control for regulators, but if licences are too restrictive it can be difficult for producers to respond to challenges or opportunities. Specification of a single species on a shellfish site relying on natural spat settlement, for example, would limit harvest of other edible species, that in some years could dominate. Some stakeholders in Norway have argued there is a need for more flexibility, and it should be easier to make adjustments, for example, moving moorings if the suitability of a site changes, or if the current set‐up potentially affects fish health and welfare.^72^

Licences are obtained through different routes depending upon the relevant jurisdiction(s), and the process of applying for a licence can take a considerable amount of time. For some jurisdictions, practicalities such as having to submit paperwork in person rather than online or by post adds further time burdens and have been highlighted as issues in the past.^31^ Some companies have in‐house expertise, while others must hire external help. Other delays can occur due to complexity for those involved in the decision‐making process, with increased need for data collection, review by relevant authorities, changing circumstances for applicants, and difficult judgements. Literature evidence of timescales is limited, often because they are case‐specific, but could be anywhere from a few months to years. For example, Suplicy et al.^34^ stated it took up to 10 years for authorization to be granted to establish a marine fish farm in Brazil. An example of time issues and the complexity within a licensing system that has well‐developed procedures is illustrated in Box 1.

BOX 1 **An example of the length of time required along with the legal complexity from a marine finfish licence application in Ireland**

In 2021, the industry press reported that Irish authorities had approved the first new marine fish farm site in 17 years, 10 years after the initial application.^73^ Marine Harvest Ireland (now Mowi Ireland) applied for the licence in 2011 which was granted in 2015. Subsequently, 14 organisations (including the applicant) appealed the decision (to the Aquaculture Licensing Appeals Board [ALAB]) under a range of different issues. The granting of the aquaculture licence was reaffirmed by ALAB in July 2021 following extensive review in addition to an oral hearing. However, in September 2021, Inland Fisheries Ireland, the statutory body responsible for freshwater fisheries management (and a State Agency) along with environmental NGOs, challenged the decision‐making process of ALAB by requesting a judicial review.^74^ The case is scheduled to be heard in the High Court in February 2023, 12 years after the initial application. It is noted that any decision resulting from Judicial Review proceedings could still potentially be subject to challenge in the European Court of Justice.

Appeals are an important part of accountability, ensuring that decision‐makers are answerable for their actions and supporting a transparent process.^44^, ^75^ Increasingly, public consultation has a formal role in planning applications, albeit with varying levels of implementation across different agencies and geographic areas.^76^ This means that supporting and opposing views should be heard during an application process^77^ and considered in the final decision in an attempt to reduce appeals at later stages. In the Irish case (Box 1), even the company applying for the licence submitted an appeal due to clauses in the licence that did not account for future technological developments.^73^ As aquaculture can be a fast‐moving and highly innovative sector, this highlights that it can be difficult for regulations to keep up.^30^

One of the other challenges for authorities is determining a fair value to place on the use of space and resources. A more detailed example for Norwegian salmon is given in Box 2. Aquaculture benefits from natural resources, but it also provides a range of social benefits, such as a source of income and jobs for local and national communities.^75^ However, in some locations, such as the Caribbean, economic rewards from other activities, such as tourism, may be more attractive.^78^

BOX 2 **Debate over resource rent and area taxation of the Norwegian salmon industry**

In Norway, there has been much debate about resource rent or area taxation of the salmon farming industry.^48^, ^79^ The Norwegian salmon industry has expanded considerably in recent decades and has become very profitable. Many, including municipal and county politicians, argued that the industry should provide more compensation to communities for the use of coastal space. A municipal area tax was proposed in the early 2000s. In 2009, it was decided that the value of the physical fish farms, but not of the fish in them, could form the basis for municipal property taxation.^80^ However, the tax income from this was very limited, and as the industry's profit margin continued to grow, the call for municipalities to receive higher tax receipt continued. Also, the state became more interested in the high profit margin in the industry. Between 2002 and 2013, all new commercial licences had been sold at a fixed price, but in the 2013 licensing round some of the licences were auctioned out,^58^ making it evident how valuable the licences were. From 2017, with the introduction of ‘the Traffic Light System’, auctions became the default allocation mechanism, and a share of the proceedings were put into an ‘Aquaculture fund’ for further distribution to municipalities and counties.^80^ This action transferred considerable funds from industry to state, municipalities and counties, more than six billion NOK (ca. 600 Mill. €) over the years 2017–2020. However, the future income from this approach was uncertain. It would depend both on how much new production capacity would be auctioned out, which, under the ‘Traffic Light System’ (discussed in Section 2.3), depends on the salmon‐lice situation in the salmon farms, and it would depend on the fish farmer's willingness to pay for new capacity. The government, therefore, appointed a public commission to consider alternative schemes for taxation of the industry, including ground rent taxation and especially ways to ensure income to the municipalities.^81^ The majority of the commission proposed a ground rent tax. The industry countered by saying they provide many benefits to local and national economies, and increased taxation could lead to the industry moving to other areas.^79^ A ground rent tax was not introduced, but a production tax of 0.40 NOK/kg of salmon produced has been levied from 1 January 2021. This will be shared amongst the municipalities that have salmon farming. The municipal share of the proceedings from auctioning of licences will be reduced. Clearly, high licensing costs can be a deterrent for industry investments, but it is the total tax package that matters. High uncertainty about future taxes or licensing costs will limit industry investments. Despite these recent major changes in the tax and fee system for salmon farming in Norway, both the right‐wing government leaving office in 2021 and the centre/labour government from 2022 have proposed new taxes or tax rules for salmon farming, including a ground rent tax,^82^ that will increase the financial burden on the industry if they are introduced.

The length of time for which a licence is granted varies between countries, although usually they are valid for several years. Producers may see a short licensing period as a risk if they have a significant financial outlay in the early years and rewards are not available immediately, whereas a long or perpetual licence may encourage investment, innovation and best practices.^44^ However, regulators have more control if renewals are required^44^, ^83^ because it allows intermittent assessment of progress, impacts, and operation. Any renewal process must be efficient as delays in processing renewals results in a high level of uncertainty for producers that may suspend larger investments until the licence is renewed.

Coastal governance is not a static process, and policies and regulations will, and should be, updated over time as new information becomes available. However, this may have implications for existing operations, and an example given by Rennie^84^ described a change in regulation that affected mussel farmers' rights to renew licences in New Zealand. Aquaculture is a business, and companies will make plans in line with the existing regulations; inequality can emerge if regulators change the system in a way that does not apply the same rules to all. This may occur when farms have obtained formal licences and begin operating before specific regulatory requirements have been established, and so, are not subject to the same rules as new applicants. Another scenario is when production areas are reclassified and then new rules are applied retrospectively to existing farms, as occurred with introduction of the Habitat Regulations Assessment (HRA) which impacted Pacific oyster farms in England and Wales, limiting expansion and innovation. Producers and regulators both have to consider the implications of changing policies, regulations and legislation.

A disconnect in timescales and requirements between licensing and funding and investment, can create challenges for aquaculture businesses. In some countries, aquaculture development can be supported through private and/or public investments, and this support can be at local, national, or regional levels, as in the European Union.^85^ However, without an active licence, it can be difficult for aquaculture producers in some countries to obtain capital investment or grant funding. Renwick^70^ highlighted the case of Irish oyster farmers who under the national legislation must have a licence to access business development grants. Grant schemes are often only available for limited timeframes, and it can be difficult to get a licence within the required time. It would be an improvement if grants could be at least provisionally granted dependent on the applicant getting a licence. The Irish oyster farmers believed that delays and challenges in obtaining or renewing licences also put them at a competitive disadvantage.^70^ The uncertainty of the outcome of licence applications and renewals, together with the length of time required for a decision to be reached, have been highlighted as some of the factors affecting the mussel sector in the EU.^86^ Sometimes farms will have been established before legislation is in place, and are in effect unlicensed, or they have a level of authorisation but no security of tenure. Unlicenced farms can also occur where there are conflicts over who owns the areas and who has the governance rights over the space and natural resources. Fox et al.^87^ noted the case of Lough Foyle, where there is jurisdictional ambiguity between Northern Ireland and the Republic of Ireland over ownership of the coastal inlet. Consequently, there is currently no regulatory mechanism for oyster aquaculture in the Lough, which has resulted in the installation of thousands of un‐licenced oyster trestles on the foreshore.

In some locations, even if a licensing and regulation framework is in place, aquaculture sites may be established illegally, without going through the planning process. Illegal development of aquaculture can have negative implications for carrying capacity, impact other users of the environment and undermine the planning and regulatory process. For example, Peng et al.^88^ highlighted that overcrowding of unlicensed oyster farms is an issue in some areas in China, and they suggested a need to reform the licensing process and the use of zones to manage development more sustainably. Earth observation technology can be used together with national records and databases to monitor unlicenced aquaculture farms,^89^ and authorities then can decide what action to take. However, this action will depend on resources and enforcement power.

### 2.3 Competition for space and marine spatial planning

It is widely recognised that coastal areas are becoming increasingly crowded with a range of different activities, and suitable space for aquaculture is often limited.^7^, ^90^, ^91^ Competition with other resource users for finite space can create tension and contribute to negative perceptions of local developments, and for the wider sector.^28^, ^32^ The (lack of) social acceptability (social licence) of aquaculture is a major issue that threatens future growth of the sector,^8^, ^76^ but is recognised as an increasingly important aspect for future developments.^92^, ^93^ Krause et al.^94^ stated that many aquaculture developments do not adequately consider the social dimensions and suggested there was a need for more public participation in aquaculture planning and licensing. Conversely, some stakeholders have suggested that governance structures that show preference to other coastal users can lead to less‐than‐optimal sites being used for aquaculture, which may have consequences for fish welfare^72^ and industry growth.

Sustainable development of coastal and marine waters requires a balanced approach where different stakeholder views can be considered in a fair and equitable manner. Different stakeholders have their own perceptions of the concept of sustainability, and views on future aquaculture development are often complex.^95^, ^96^ There is a need to understand the drivers behind different views and expectations across stakeholder groups and demographics, including local communities and the public.^97^ Both industry and authorities should be interested in measures that can increase the social acceptance or ‘social license to operate’ for the industry. This has in some cases been found to depend on peoples' perceptions about the fairness of distribution of benefits and burdens from industry activities, about the governance system, and the communication and relationship between industry actors and society (e.g., Sinner et al.^98^). Measures to increase the legitimacy of the governance system^99^ then can be relevant. It could also be that lack of information or misperceptions are behind views and expectations. It may be especially important to engage with Indigenous people,^50^ as highlighted earlier, as they may hold veto powers.

Marine spatial planning (MSP) is promoted as a way of planning and managing different resource users to minimise conflict.^100^, ^101^, ^102^ As an iterative and participatory process, MSP allows different views from sectors/users on ecological, social and economic objectives to be discussed by a range of stakeholders with decisions on how to use space and natural resources within an area to achieve defined objectives. Thus, in theory, it should increase communication between different stakeholder groups, optimise use of space and reduce tension.

Effective MSP can require a considerable amount of time and resources to develop and implement.^103^ Collie et al.^104^ reviewed 16 existing plans throughout the world and found that they took at least 1.5 years to develop, and costs ranged from <US$500,000 to almost US$5 million per year of planning, mostly funded by national or state governments. Absence of policies and regulatory support is a constraint.^105^ For example, Chen and Qiu^106^ suggest that aquaculture development in Taiwan would benefit from more coordination of planning and management in the coastal zone, but lacked administrative and legal instruments to facilitate this.

In contrast, all member countries in the European Union (EU) have legally committed to establishing marine spatial plans as key to meeting the objectives of the Marine Strategy Framework Directive (MSFD) and delivering the EU's Blue Growth Strategy for healthy and productive seas, in which aquaculture is one of the key sectors.^107^, ^108^ To facilitate the development of effective plans, the EU has provided member states with support through legal frameworks, financial assistance, guidance and training, and facilitated transboundary co‐operation.^108^ There are also regional efforts to support MSP such as the work done by the Regional Commission for Fisheries (RECOFI) for the Near East region.^102^

Knowledge gaps and uncertainties can be barriers to fully implement a marine spatial plan. Deidun et al.^101^ noted that a lack of data had prevented the establishment of marine spatial plans in Malta, but also recognised that this was not sufficient justification to maintain the status quo; it is possible to learn from experiences in similar locations and collect data to update and revise plans in an iterative process. This is a key point, as a marine spatial plan is not an end in itself and it needs to be regularly reviewed, as they can become outdated.^104^

Most MSP tools are static and only provide a snapshot of existing uses,^104^ and simple maps may not tell the full story.^109^ There has been a move towards more real‐time and adaptive management of marine systems,^110^ but there are few examples of plans that incorporate dynamic changes.^111^ MSP should be an iterative and evolving process,^104^ and this includes use of new data and approaches when they become available. This is particularly important for a dynamic sector such as aquaculture where technological innovations, changing environmental conditions and emerging biological challenges can require frequent policy and regional changes.^30^ For example, it was acknowledged in Norway that many existing marine spatial plans needed to be updated as the suitability of locations for aquaculture had changed due to revised environmental and veterinary regulations, as well as the introduction of new production technologies.^112^ It may become increasingly important as climate change affects water temperatures and system dynamics, impacting where species can be grown.^113^

AZAs, that is, dedicated areas where aquaculture production is prioritised, are a way of incorporating aquaculture into MSP.^91^ Defining where a specific activity can take place is an important aspect of MSP, but this must be done systematically, based on evidence of suitable environmental conditions for aquaculture production and dialogue with other resource users and stakeholders. Zoning should have some degree of flexibility to accommodate the requirements of different existing and emerging species and systems, as well as potential innovations.^40^ Suitable locations for individual aquaculture sites or zones can be identified using geographic information systems (GIS) and spatial modelling to find areas that fit the specified criteria,^114^, ^115^ such as: locations that have the fastest growth potential for shellfish,^116^, ^117^, ^118^ sites that can be used for finfish cages^119^, ^120^, ^121^, ^122^ or areas where farm infrastructure would have minimal visual impact.^123^, ^124^, ^125^ A key advantage of spatial modelling is the ability to combine data from a range of sources across disciplines, enabling identification of suitable sites or zones based on biological, ecological and socio‐economic factors.^114^

Many GIS studies on marine aquaculture site selection^116^, ^117^, ^126^ integrate other marine users and activities, such as transport, military use, energy production, tourism and mining, and can allocate buffer zones around activities into the models to indicate areas that may not be available for development. In considering other activities, these models not only aggregate suitability of the zone/area for all, or specific, types of aquaculture production, but also availability, something that is particularly important in crowded coastal areas. Additionally, there is a wide range of MSP models and tools that have been developed to evaluate trade‐offs between different activities, and these have been reviewed recently.^127^, ^128^ A major strength of using models when preparing marine spatial plans is that a range of alternative scenarios can be simulated to identify the most appropriate development options for an area in a cost‐effective way. For example, Coccoli et al.^129^ simulated a range of potential changes to fishing activity along the Basque coast with the introduction of a new aquaculture site and were able to identify options for fishermen to reallocate effort to alternative areas, and thus co‐exist with aquaculture.

Zones, or management areas, can be used to coordinate activities such as disease management/treatments or stocking/harvesting, but zones used for planning purposes may not necessarily conform to biosecurity requirements.^40^ Some regulators have chosen to regulate production levels based on specified conditions within an area. In Norway, the coastline has been divided into 13 production zones where salmon aquaculture production capacity is regulated based on how sea lice levels in the fish farms affect mortality of wild salmon. Introduced in 2017, and known as the ‘Traffic Light System’, production areas are categorised as green (can increase production capacity), yellow (production capacity should be maintained) or red (production capacity should be decreased).^130^, ^131^ However, focusing on one single indicator can be an oversimplification for a complex sector like aquaculture and can lead to unintentional effects. To stay within the strict sea lice limit, farmers have used anti‐lice treatments which, in turn, had negative consequences for fish welfare and mortality, as well as for company costs/profitability, and without seeming to reducing the sea‐lice infestation pressure on wild salmonid populations.^130^ The Norwegian Traffic Light System is controversial, with mixed opinions between stakeholders.^131^, ^132^ In 2021, a group of farmers from one of the production areas took the government to court on their belief that the system was flawed and unfair.^133^, ^134^ Although area‐based management is intended to encourage stakeholders to work to a common purpose, making decisions using indicators at an area or regional level leads to questions of fairness; for example, salmon farmers operating within limits can still be penalised for the actions (or lack of actions) of others.^132^ In recognition of this issue, farms that fulfil strict criteria regarding lice levels and anti‐lice treatments have been allowed to expand capacity regardless of the traffic light colour designation of the production zone where they are located. Still, concerns over fairness can undermine both the regulatory system and its legitimacy amongst all stakeholders and the wider public^131^, ^135^; this issue is also relevant to the spatial planning of potential aquaculture areas.^99^ In spatial planning, fairness requires consistency in decisions, with the same rules applied to all. Using examples of Strategic Environmental Assessments (SEAs) in Norway, Mikkelsen et al.^99^ showed that it can be difficult to ensure consistency in decision rules as local values on use of an area will vary, and decision‐makers may interpret such values differently depending on their experience and familiarity with the location. External consultants with little connection to an area may be more consistent in applying decision rules in the SEA, but the decisions may not reflect the values of the community.^99^ So even if a decision appears to be consistent and fair on paper, if local values are not sufficiently considered the local community may feel disenfranchised, and their trust in the planning process may be reduced.

Local‐scale (sub‐national) planning has an important role in gaining social acceptance of aquaculture, and building relationships with local communities can reduce conflict, increase trust, and improve perception,^136^ which in turn enhances the support for the industry, facilitating future development. In a study of public comments from Scottish finfish aquaculture planning applications, Billing^77^ found that many supporting comments referred to actions of the applicant, trust in how they operate, and the applicants previous record of compliance. Some countries have implemented non‐statutory local‐scale planning and management initiatives to support more coordinated use of resources amongst different stakeholders.

In Ireland, the Co‐ordinated Local Aquaculture Management Systems (CLAMS) and Single Bay Management (SBM) practices are mechanisms for local participation in decision‐making by identifying and resolving conflict between aquaculture and other activities and stakeholders.^27^ In each case where the CLAMS process is applied, a plan is established that fully integrates aquaculture interests with relevant national policies as well as SBM practices, interests of other users, Integrated Coastal Management Zones (ICZM) and County Development plans. The CLAMS process is driven by the aquaculture producers in each area.^137^ Co‐ordinated planning and management require time and resources, as well as commitment from all involved if they are to be effective. Although CLAMS is non‐statutory and a voluntary scheme, dedicated Liaison Officers are financed by central government to help with facilitation and to prepare plans for each of the coastal bays/regions that have CLAMS in place.^138^ CLAMS was originally developed for salmon, but was then extended to other species, although Renwick^70^ noted that it was not mentioned during their interviews with oyster producers. Carr^27^ suggested CLAMS could be strengthened, including more involvement with wider stakeholder groups, to improve marine spatial planning processes. A nationwide review of CLAMS by the Seafood Development Agency (BIM) is currently underway and many of these aspects will be considered.

In many European counties, Fisheries Local Action Groups (FLAGs) are used to support communication and develop partnerships between stakeholders using the same coastal areas.^76^ It is also important to note that, given the complexities and trade‐offs involved in MSP and management of natural resources, there may be a mismatch between priorities at different spatial scales. Mongruel and Pérez Agúndez^139^ discussed how local‐level planning and management of shellfish production within the French Bay of Mont‐St‐Michel had avoided overexploitation of the natural resources by collective management decisions in the Bay that had decreased overall exploitation intensity, and in doing so, led to job losses and fewer employment opportunities. Thus, local‐level socio‐economic practices did not align with national policies that aimed to maximise people's benefit from the resources by supporting as many jobs as possible and contributing to public budgets via land taxation.

### 2.4 Emerging species and diversifying marine aquaculture production

The word ‘emerging’ is used here to refer to aquaculture species and systems that are not commonplace within an area, rather than being completely novel to global aquaculture. Seaweed production, IMTA, nutrient and carbon offsetting with aquaculture, offshore aquaculture and multiuse platforms received a notable amount of attention in the literature, and the key points are highlighted in this section.

#### 2.4.1 Seaweed

Seaweed (macro‐algae) farming has long been commonplace in Asia where it dominates aquaculture and seaweed is produced in large volumes.^140^ In recent years there has been enhanced interest in seaweed farming across the world for a range of ecosystem services including food provision and carbon offsetting.^141^ Seaweed aquaculture can help to reduce greenhouse gas emissions from food systems; for example, use as a replacement for some terrestrial‐produced human food products, use as animal feed (particularly for ruminants) and in some cases, it may be possible to use seaweed as an alternative low‐carbon footprint material (e.g., bioplastics),^142^ as well as reduce need for harvesting from the wild.

At present, there is very limited seaweed production in Europe and other parts of the world outside of Asia and so is considered a new and emerging sector in these regions.^143^, ^144^ However, initiatives such as ‘Seaweed for Europe’,^145^ the ‘Safe Seaweed Coalition’ (https://www.safeseaweedcoalition.org/) and the upcoming ‘EU4Algae’ (the latter part of the EU From Farm to Fork strategy)^146^ highlight the interest in boosting production (and consumption) of seaweed in the EU (with a potential market value of €9.3bn and production of >8 million tonnes of fresh weight by 2030^145^).

One of the bottlenecks for the development of the seaweed sector in many countries outside of Asia is that licensing and regulation often are designed for finfish and shellfish operations,^147^, ^148^ and absence of a clear framework for seaweed production creates confusion for applicants and deters investors.^149^ Wood et al.^147^ suggested that regulators had not established a clear regulatory process to set up seaweed farms in the United Kingdom, and this has now been identified as a critical need in the English Aquaculture Strategy to enable the development of seaweed aquaculture.^14^

Knowledge gaps and insufficient evidence base (e.g., on entanglement risk for marine birds and mammals in the farm structure; potential loss of seaweed biomass from the farm) create challenges for authorities in establishing whether proposed seaweed farming activities may result in negative environmental impacts,^144^, ^147^, ^148^ potentially requiring additional and overly stringent data requirements for the applicant. Gjertsen et al.^150^ surveyed coastal planners in Norway about the potential for algae farming, and 85% of the municipalities suggested they had unsatisfactory knowledge of the topic. While authorities may not need a high level of expertise in a particular species and production system, they do need an understanding of certain subject areas to ensure that appropriate decisions can be made on aquaculture development. Thus, as discussed by Gjertsen et al.,^150^ lack of planning competence could be a barrier to the emerging industry. Stévant et al.^151^ also reviewed the state of the art and some of the knowledge gaps and suggested that researchers and authorities in Norway should work together to establish an evidence‐based regulatory framework for the sector. This also applies to other countries that are seeking to build a seaweed farming industry as an emerging sector. Although seaweed aquaculture is relatively new in countries like Norway, it is also important not to reinvent the wheel. Knowledge can be translated and transferred from countries with a long‐established seaweed farming sector like China.^152^, ^153^

#### 2.4.2 Integrated multitrophic aquaculture

IMTA generally refers to the integrated production of extractive species, such as seaweed and shellfish, which use waste nutrients from other farmed species such as fed finfish.^154^ Although IMTA has been practiced in China and other Asian countries for a long time, albeit not necessarily under the term IMTA and often incidental rather than deliberately planned,^71^ in most other parts of the world the concept has only gained popularity in the last decade or so. IMTA is promoted as a way of diversifying farm production, by adding additional species, reducing waste impact through nutrient recycling^154^ and providing financial benefits through product diversification.^155^ Alexander et al.^36^ suggested that the bioremediation potential of IMTA could be an incentive for development. However, demonstrating a trophic connection and then bioremediation service within an open‐water IMTA is difficult, especially at a farm‐scale.

In comparison to closed land‐based IMTA systems, coastal or open‐water IMTA is more challenging due to the dynamic nature of the marine environment and uncertainties about the transfer of nutrients between the grown species.^156^ Farm management practices, site layout, and physical environmental characteristics all influence the dispersion of solid and dissolved nutrients, and environmental and biological factors also influence uptake by extractive organisms.^157^ This makes setting general guidelines difficult as a range of combinations of species and production techniques could be involved, for example, suspension‐feeding bivalves,^158^ organic deposit‐feeders such as sea cucumbers^157^ and inorganic nutrient‐extractive species such as seaweed.^159^ Not all combinations of species and trophic groups will be appropriate in every area. For example, Christensen^160^ expressed doubt that commercial‐scale salmon‐seaweed IMTA could be developed in the Faroe Islands due to a seasonal mismatch in nutrient loading and uptake between species, as well as a lack of available space. Accordingly, during planning and licensing for IMTA, there needs to be consideration not only of the suitability of location for each species, but also practicalities of how to define environmental interactions within an integrated system.

The literature highlights poor or absent licensing and regulation systems as an obstacle to IMTA development in several countries, for example, Cyprus, Denmark, France, Ireland, The Netherlands, Spain, and Korea.^46^, ^161^, ^162^ This is the case when IMTA is at pilot‐scale and regulatory frameworks are required for IMTA to move towards commercial‐scale production. Existing regulations tend to focus on single species production and are different for fish, shellfish and seaweed,^151^ which makes it difficult (or not legally possible) to establish an IMTA site. In recent years, several countries have amended their aquaculture policy strategies to focus on the need for improved environmental sustainability (e.g., Scotland, Cyprus and Norway), as well as diversification and innovative technology (e.g., Italy and Ireland).^36^ This presents an opportunity for the development of IMTA, but still, stakeholders recognise the lack of legislation and licensing processes for IMTA^163^ that could in part be driven by poor public perception and opposition from environmental groups.^164^ Policies and legislation need to address persistent concerns around commercial‐scale IMTA that include disease transfer, fish health and food safety, balanced against the likely environmental benefit, even if not entirely measurable at this point. Moreover, the development of policies that recognise IMTA products would help overcome barriers that underpin the commercialisation of IMTA and sustainable development of aquaculture.^36^ For example, Ellis and Tiller^165^ point to uncertainty about whether IMTA products are allowed for human consumption in Norway. In addition to food safety, considerations for social acceptability^166^, ^167^ and certification should be dealt with before significant investments in developing sites and production can be expected.

Regional‐scale IMTA occurs at a waterbody level, such as designation of a fjord or coastal bay as an ‘IMTA’ location.^168^, ^169^, ^170^ In regional systems, production does not have to occur in proximity to the fed species but within a defined area, at a distance between farms, and consideration is given to whole‐bay nutrient fluxes as a measure of environmental potential for the IMTA system, without need for integrated sites, although this could still be enacted where regulation allows or is changed to allow it. In areas where regulations have distance restrictions (example in Box 3), and as Ellis and Tiller^165^ note, it may be more feasible to consider regional‐scale IMTA, in a cooperative manner involving many farm owners. Even though it is not designated as such, in some areas, there is unintentional regional‐scale IMTA, with fed and extractive species co‐located, with extractive species benefitting from dissolved, and possibly particulate, nutrient inputs from fed species, albeit the farming activities are entirely separate. A balanced regional ecosystem approach may be a means to show benefits as is more typically done for particular species (e.g., van der Schatte Olivier et al.^171^) and generate political support to drive regulatory changes.^165^ To fully implement a regional approach to IMTA, there may be a need for semi‐formal or formal management structures, supported by policy and regulations where appropriate. This is important, as several different companies may be involved so there is a clear need to establish rights and responsibilities, similar to mechanisms like CLAMS (see Section 2.3) that support co‐ordinated management of an area. This management approach would require adequate resourcing to implement.

BOX 3 **Example of distance considerations for IMTA siting in Norway**

In Norway, regulations state that an aquaculture facility must not be placed so that it poses an ‘unacceptable risk’ for the spread of pathogens. In the consideration of this issue, the distance to watercourses and other aquaculture facilities, as well as aquaculture species, production type and volume, shall be especially emphasised. The formal regulations do not state specific distances (e.g., in km) (with one very special exception). The Norwegian Food Safety Authority's guidelines for handling of aquaculture applications does, however, state recommended distances.^172^ According to this recommendation, shellfish farms cannot be close to other aquaculture facilities, with 1.5 km minimum distance as a main rule, unless there is an agreement of co‐location or joint operation. The guideline also has a separate point on IMTA. Permits are normally given only to one species for each location, in accordance with the Aquaculture Act, but exemptions may be applied for^173^ and have been granted for IMTA for salmon and shellfish. The Norwegian regulations probably need to be changed to facilitate significant expansion of IMTA.^165^

The lack of clarity in the existing legislative frameworks, and the different policies and regulations that govern IMTA at the EU and national levels, pose challenges to cohesive implementation across Member States.^36^ EU mechanisms (e.g., the EU Aquaculture Advisory Council) are key to communicate pressing issues for IMTA development, and to support the effective implementation of policies and legislation across the EU. Where regulations do not enable IMTA development, amendments to public policy may rely more on stakeholders to advocate for IMTA and influence policy makers and public perception to advance IMTA regulations, for example, in Norway.^165^

IMTA development relies on licensing systems that permit multiple species and activities, and on policies and legislation that enable multitrophic farming practices with an ecosystem management approach. Regulatory and legislative changes need to be backed by agreement to permit demonstration sites/zones, and consideration of technical, biological, and economic viability at commercial‐scale production. Investment in IMTA depends on financial sustainability and research‐based concessions for an assessment of specific criteria, such as site selection and market demand.^165^ While IMTA is considered an option for sustainable aquaculture development, particularly where nutrient input exceeds critical ecological thresholds,^165^ investment will depend on conditions of transferable research licences to commercial ones once the criteria for financial risk have been met.^174^

#### 2.4.3 Offsetting with aquaculture

Extractive aquaculture refers to production of non‐fed species, such as bivalves and seaweeds, that rely on the natural environment for their nutrition. When grown in large quantities, like an aquaculture farm, extractive species influence nutrient dynamics within the wider ecosystem, and have an important role in the consumption and movement of energy within marine systems. Bivalve molluscs, for example, can filter large volumes of water and remove excess phytoplankton and enhance denitrification in sediment.^175^, ^176^, ^177^ Therefore, extractive species potentially can mitigate water quality issues such as eutrophication.^178^, ^179^ It is also suggested that harvest is a measure that will remove nutrients from the marine system.^180^, ^181^ To this end, nutrient offsetting by extractive aquaculture is proposed as a means to reduce nutrient levels and offset some of the impact of other nutrient‐contributing activities.^182^ The concept has gained attention as an approach that could be used in areas such as the Baltic Sea,^183^ where eutrophication is identified as a major problem.^184^ However, as with other forms of aquaculture, not all locations will be available and some sites will be more suitable than others, so GIS models can be used to identify areas that are suitable for extractive aquaculture, hydrodynamic and biogeochemical models can be used to explore nutrient flows, and bioenergetic models can be used to simulate nutrient uptake and use by extractive species to evaluate the potential for improved water quality by nutrient removal.^185^, ^186^

Extractive aquaculture solely for offsetting purposes may be considered advantageous in areas that would not normally be considered optimal for food production, for example, areas where land runoff introduces pollutants or bacterial pathogens,^187^ such as urban estuaries.^188^ However, in such contexts development and operation of an extractive aquaculture site will require resources. Without products that could be sold for human food purposes, Rose et al.^189^ noted that nutrient remediation alone may not offer sufficient incentives for many extractive aquaculture producers, and therefore, to recuperate costs, producers may need to look for other market opportunities. For example, bivalve shells could be used as a source of calcium carbonate for industrial or manufacturing purposes.^190^ A planning and licensing system that has been established based on aquaculture for food provision may also be a bottleneck, as there may be a presumption against development in particular locations due to food safety concerns. To promote improvement of water quality, governments could also subsidise operators of extractive farms that would not be able to see their products for food. Zheng et al.^153^ suggested that government incentives may even be needed to grow seaweed species of lower economic value, but higher remediation potential in China since farmers tend to prioritise economic gain. Marine spatial plans may have to demarcate different areas for aquaculture based on food or non‐food purposes. In some cases, there may be a need to choose between different ecosystem services, and decision‐makers may have to look at trade‐offs between the respective services to identify the best use of space and resources.^191^ Given the different economic strategies and management practices that would be employed for food versus non‐food sites,^189^ producers would also have to decide what is economically feasible, especially if regulators impose specific conditions on a site.

The potential role of aquaculture in carbon offsetting is also receiving considerable attention as a potential climate‐change mitigation measure.^192^, ^193^, ^194^ For example, seaweed biomass can be used to replace food, feed, fertilisers and materials associated with higher greenhouse gas emissions, as well as for production of biofuels.^142^ Some studies have suggested that seaweed could be grown at large scale and then sunk to the deep sea to temporarily lock carbon away from the surface,^193^ but there are many uncertainties within this approach and more research is required.^142^

A range of schemes have been suggested for payment for ecosystem services (PES),^195^, ^196^, ^197^ although valuation of ecosystem services is not always financially focused.^198^ Nevertheless, of particular interest to many in aquaculture is the use of certified credit schemes, where organisations purchase credits from the aquaculture producers to compensate for, or offset, their own emissions or environmental impacts, whether they are nutrients or carbon.^169^ Unlike IMTA, where the extractive species need to be in relatively close proximity, aquaculture sites used for offsetting do not need to be in the same location, or even country, as the activities they will offset. However, offsetting is complex, and there are many knowledge gaps that still need to be addressed, particularly in the case of carbon.^142^, ^199^ One of the challenges is that assimilation depends upon the species, location, culture method, and time of year.^200^, ^201^, ^202^ Therefore, it is difficult to assign simple fixed values, as this approach may over‐ or under‐estimate the nutrients or carbon removed by a particular operation. Understanding the transfer of nutrients or carbon from the environment to the farmed species is of fundamental importance for accounting purposes, but it is also important to determine the fate of the nutrients and carbon to ensure these are removed from the system and not just converted into another form and released back into the environment (e.g., respired or excreted). These challenges are not unique to aquaculture systems; carbon offsetting has largely been limited to terrestrial forests, as coastal and marine ecosystems are included in fewer than 10 projects worldwide^203^ and to date only include mangrove forests. Issues of the provenance and fate of carbon into and from marine ecosystems were identified by Shilland et al.^204^ as bottlenecks to the certification of seagrass meadows under carbon‐offsetting schemes. Moreover, while reduction may be the overall aim, excessive depletion can affect other ecological processes and the wider ecosystem, so carrying‐capacity assessments will be important for establishing the optimal size of farms.^205^, ^206^

Even if the proposed production system intends to provide wider benefits such as improved water quality, community acceptance can still be an issue.^207^ Any development will use space and resources, and local communities can have strong opinions on how, and if, an aquaculture site should be established.^206^, ^208^ Petersen and Stybel^207^ described a situation where the local community had a high level of distrust for a mussel farm even though the test site was installed to reduce nutrient levels. In that location, community opinion had been affected due to poor perception of aquaculture within the area, and people were sceptical about the environmental benefits.^207^ To increase trust, developers should work with local communities to gain insights into their concerns and expectations.^206^ In a different location, Petersen and Stybel^207^ found stakeholders were more accepting of the mussel farm and recognised the improvements in water quality. This was partly because there was no history of distrust with the producers, but also because the community had seen the benefits from the test site.

#### 2.4.4 Offshore aquaculture

Planning, licensing, and governance issues associated with offshore aquaculture have been a hot topic in recent years.^117^, ^209^, ^210^, ^211^, ^212^, ^213^ This is unsurprising as offshore aquaculture is gaining considerable attention as a potential solution to increase marine aquaculture production outside of coastal locations, particularly in the United States. However, the absence of a strong planning framework is considered a major bottleneck to offshore development in the United States.^41^, ^209^ The United States is not alone in this regard. As highlighted by Davies et al.,^93^ few countries have governance structures in place that specifically mention ‘offshore’ production. One of the issues may be the lack of an accepted and uniform definition for offshore production and a range of perceptions over what offshore actually means, in addition to a preference to use other terms such as open ocean, exposed and highly dynamic sites.^214^ Still, the most obvious reason for the lack of developed regulations is the lack of widespread commercial technologies for offshore aquaculture production, with many aspects still under development.^215^, ^216^

Existing planning and regulatory frameworks that have been developed for inshore and coastal locations may not be appropriate for offshore production.^209^, ^214^ As aquaculture moves further offshore, production technology may change,^216^ and environmental conditions and interactions with aquaculture may be different.^213^, ^217^, ^218^, ^219^ Environmental regulatory quality standards that are based on inshore environments may not be appropriate for offshore aquaculture.^214^ Furthermore, the authorities responsible for planning and regulation of offshore environments may be different to those inshore. For example, in the coastal zone of Norway there are municipal, regional, and state authorities involved in decision‐making, whereas beyond the coastal areas it is only state actors.^220^, ^221^ Arguably, dealing with fewer number of authorities may streamline the process. In the case of the United States, Lester et al.^222^ suggested there was a need for a more streamlined approach to offshore aquaculture regulation in federal (national) waters, in contrast to the disjointed and fragmented state‐level regulations for nearshore coastal waters that involve multiple state and federal agencies, and are considered a bottleneck to aquaculture development. Regarding international waters, though most aquaculture is likely to stay within national exclusive economic zones (EEZ) for the foreseeable future, Percy et al.^223^ note there is no international law related to aquaculture outside of these areas.

There is also a need to ensure that regulatory authorities have the capacity and resources available to implement and enforce regulation.^212^ In a study by Fairbanks,^209^ stakeholders highlighted that development of offshore mussel farming in New England, the United States, had been slow due to unfamiliarity with offshore aquaculture amongst agencies which often led to precautionary rejection of applications. Stakeholders felt that the different authorities involved often had a narrow focus and lost sight of the ‘big picture’, which in some cases led to trivial or non‐existent issues restricting development.^209^ Lack of knowledge and experience amongst authorities was also cited as a reason for delays in obtaining the relevant permissions to establish an offshore mussel farm in the south of England, a case that is described in detail by Corbin et al.^224^

#### 2.4.5 Co‐location and multiuse platforms

Co‐location and multiuse platforms or floating artificial islands, where marine aquaculture is combined with other activities such as offshore energy production, are sometimes suggested as a way of reducing conflict, sharing resources and optimising use of space.^225^, ^226^, ^227^, ^228^ In multiuse platforms, activities are integrated within the same structure(s), whereas co‐location as a broader concept can involve multiple activities sharing the same space (and infrastructure such as shared electricity) without being physically connected to each other.^229^

Sharing space and resources is considered by some to be a way of increasing social licence to operate.^100^ However, there can be disagreements over how to use space, even if it could serve multiple purposes. Wever et al.^228^ held a stakeholder workshop about the potential for integrated offshore wind and aquaculture in the German North Sea and reported that researchers believed there was a strong political interest in developing multiuse platforms, while representatives from environmental agencies disagreed. Likewise, Van Hoey et al.^219^ reported that fishery representatives saw co‐location as a risk and were sceptical of any advantages, so a long‐term and continuous process of engagement amongst all stakeholders would be required to address concerns.

There needs to be clear benefits for all involved groups for multiuse platforms to be realised.^230^ For small‐scale aquaculture producers with limited capital there may be advantages to sharing space, infrastructure, and vessels, but for activities such as offshore wind, benefits of co‐location may not be as apparent, and government interventions may be required to encourage development.^227^, ^231^ In offshore, high‐energy environments, existing platforms may provide anchor points that would otherwise be unavailable to aquaculture.^230^

Installation of aquaculture at decommissioned oil and gas platforms could remove the need to dismantle structures,^224^ which may be attractive to platform owners as it would alleviate some of the costs associated with decommissioning.^232^ In 2021, funding was granted to repurpose a former oil platform in the Gulf of Mexico into a commercial fish farm.^233^ This project is likely to attract considerable attention from across the world as other companies wait to determine if such a development is feasible.

Understandably, co‐location and integration of multiple activities create major issues for planning and licensing as these activities have individual and cumulative needs and impacts. As highlighted by Depellegrin et al.,^234^ although the impact of individual activities is often well understood, there are many uncertainties about combined effects. van den Burg et al.^235^ suggest a precautionary approach should be taken until reliable data are available from approaches that consider cumulative impact assessment. However, van den Burg et al.^235^ also assert that more information from real‐world in situ testing is required. This agrees with other authors^234^, ^236^ who state pilot sites are essential if co‐location and multi‐use platforms are to be established. Such trials provide valuable information on environmental impacts, technical considerations and socio‐economic concerns, all of which are important for developing a robust planning and licensing system that enables development of full‐scale commercial systems, if shown to be appropriate. Operators of each activity also must have secure and clear property rights to allow for long‐term investment and clarity over use of space.^230^ Legal clarity over rights and responsibilities (such as data, insurance, licensing application time and costs) is also required. Such clarity would facilitate communication between the different users, who may be unfamiliar with what is involved in each sector, to define formal protections^237^ including long‐term licences, as their absence increases uncertainty and discourages development.^236^

### 2.5 The need to address knowledge gaps and use of decision support tools

Regulations and licensing frameworks are often underpinned by the available scientific evidence. However, as discussed by Osmundsen et al.,^30^ the dynamic nature of aquaculture and the high level of innovation can be difficult to cover fully in regulations, and this dynamism could be both a driver for, and a response to, innovation. It is important to regularly review state‐of‐the‐art information and identify knowledge gaps that need to be addressed to support sustainable development of the aquaculture sector.^238^

Planning and licensing systems can obstruct or facilitate innovation. In many countries, the planning and licensing system has been established for certain species and production systems, with little flexibility for alternatives or newer technology. In Norway, the licensing system has several different categories in addition to ordinary commercial aquaculture licences.^239^ Hersoug et al.^61^ have covered the range of special purpose licences (Broodstock, Education, Exhibition, Research and Development) for salmon farming that are available in Norway, within which there are now many potential routes to aquaculture development, and which Hersoug^239^ notes may be both a good and a bad approach. In particular, Research licences are used by research institutions to generate new knowledge and Development licences are used by companies for high‐cost and high‐risk innovations.^61^ Prioritising the allocation of ordinary commercial licences for salmon farming to organisations that gave the most promising visions for innovation had previously resulted in very limited effects, not least as the allocations were not followed up with clear demands from the authorities to report whether such promises were fulfilled.^58^ The Development licences have, on the other hand, been a major pathway to permitting trial offshore salmon farming in Norway, testing large‐scale systems such as Salmar's Ocean Rig and Nordlaks' Ocean Ship (Havfarm).^80^ Without these long‐term and large‐scale demonstrations, it would be difficult to examine many aspects of these systems.

A range of decision‐support tools exist that can be used within aquaculture planning and licensing, though there are varying levels of adoption as a formal regulatory requirement. Decision‐support tools come in many formats, from simple spreadsheets and checklists to complex computationally intensive models.^240^ Some have been developed specifically for regulation, others have been adapted for use, and there are also tools that are primarily used for research purposes. The use of models and tools for MSP, site selection and zoning (see Corner and Aguilar‐Manjarrez^241^) was covered more generally in Section 2.3, but models are also important for other aspects of planning and licensing. Identifying a suitable location or zone is the first of the two key elements, as noted previously, the other being a need to ascertain how much production can be realised.^4^ The ability to predict the potential impact (positive or negative) of a new development on the surrounding area is particularly useful for planning and licensing decisions. For example, waste dispersion models^242^, ^243^ offer insight into how finfish aquaculture may impact the area surrounding a proposed net‐pen and can be used to determine acceptable production levels.

Early environmental impact models tended to focus on the immediate area surrounding a farm, but over the last decade there has been a move toward approaches that consider far‐field (>1 or 2 km) effects, often with use of hydrodynamic models to evaluate waste transport beyond the immediate farm environment.^244^, ^245^ In addition to wastes, models also are used to simulate the potential spread of disease (i.e., parasites) and connectivity between farms and consider potential aquaculture siting implications for disease management.^246^, ^247^, ^248^ Models can provide valuable decision support for extractive species such as bivalves and seaweed that are reliant on the natural environment for food, by estimating nutrient fluxes, carrying capacity and production potential.^249^, ^250^, ^251^, ^252^, ^253^ In Northern Ireland, the Sustainable Mariculture in northern Irish Sea Lough Ecosystems (SMILE) models^254^, ^255^ have been developed to assess ecological carrying capacity for shellfish aquaculture and to support planning and management decisions. The SMILE modelling approach (Box 4) is an example of how decision support can evolve within aquaculture, if properly resourced and given the time to revise and update approaches when new information and technology become available.

BOX 4 **Description of the SMILE modelling framework as an example of how decision support can evolve in aquaculture**

The Sustainable Mariculture in Irish seaLoch Ecosystems (SMILE) model framework combines field data, experimental results, and various types of models, ranging from individual shellfish growth models to broad‐scale ecosystem models.^254^, ^256^ The process by which these models are integrated and coupled is designed to capture the essential information at each simulation scale, whilst allowing multi‐year runs which provide estimated potential cultivation of commercial species, nutrient and chlorophyll cycling, and other outputs of interest to decision makers. The complete modelling framework facilitates integrated analyses of animal‐environment interrelations affecting overall production at system‐scales, according to different temporal and spatial scenarios and accounting for conservation aspects such as the presence of wild species. Models like SMILE allow managers to examine the potential outcomes of different development options/scenarios without the social consequences of experimental implementation. The initial use of the SMILE model was to help manage the shellfish aquaculture industry, though it has evolved to focus more on ecosystem health and sustainable development of the shellfish industry. It focuses on chlorophyll *a* (Chl *a*) as an indicator of ecosystem health and examining the effect of aquaculture on Chl *a* in a system.^257^ Aquaculture species reduce the overall ecosystem phytoplankton biomass and food availability for other organisms. Annual variation within Chl *a* values (using 90th percentile figures) recorded between sampling years is calculated from analysis of historical data for each sea lough. The percentage difference between years provides a baseline value (%) of Chl‐*a* that should remain within the system for wild species.

The model framework for SMILE was expanded to include the catchment, initially for Lough Foyle during the EASE project,^256^ and currently a modified approach forms the basis for the ongoing coastal catchment models. The focus on catchment to coastal modelling is to help support management decisions at the catchment level. The SUCCESS (System for Understanding Carrying Capacity, Ecological, and Social Sustainability) model framework, includes the catchment (hydrological—SWAT), circulation (hydrodynamic—Delft3D), individual bivalve (AquaShell) and Ecosystem models (EcoWin). Collectively, these products aim to allow the definition of bay‐scale management measures, including scenario modelling, to meet the requirements of EU directives such as the Urban Waste Water Directive, Water Framework Directive (including protected areas), Habitats Directive, and Marine Strategy Framework Directive. The coupling of drainage area model outputs allows scenarios to be run investigating urban and diffuse inputs, and scenarios are used to examine best management practices in a catchment.

Many decision‐support models for planning and licensing of fish aquaculture have focused on Atlantic salmon production, likely due to its commercial importance, high value and the amount of knowledge and data available for this species under production environments. Although some models can, and have been, adapted,^258^ other species and locations will require development of new and bespoke models. For example, in the absence of appropriate existing models, Chary et al.^259^ constructed a model to simulate growth potential and waste outputs from red drum (*Sciaenops ocellatus*) farms in Mayotte in the Indian Ocean. Further models will be needed for new and emerging species and for new systems, such as IMTA. It is also important to acknowledge that model development is not an endpoint, and use of models for decision support during planning and licensing will depend on several factors. As discussed in Falconer et al.,^114^ if decision‐support tools are to be used by industry and regulators, then they must be accessible and they must have an appropriate lifespan. If models are built using specific software or routines, then they may become outdated or unusable.

In some countries, such as Scotland and Ireland, it is a regulatory requirement to use models within the planning application and Environmental Impact Assessment (EIA) or for treatment consents. Environmental models can have an important role in regulations as they provide information on potential scenarios that allow decision‐makers to assess potential effects before an action. However, it is also important to recognise the burden that specific requirements may place upon applicants, for example, expecting organisations to use complex, data‐intensive or technologically sophisticated tools may be a barrier to smaller companies, even if they are able to hire consultants to perform the work for them. In some jurisdictions, regulatory authorities may facilitate use of models in planning and licensing applications, either being fully or partially responsible for modelling, or providing guidance and training for statutory approaches. There are no uniform requirements or approaches to modelling throughout the aquaculture sector. Ultimately, some species and locations have more data available than others, which can affect use (and choice) of models for decision support.

Models and tools are dependent on suitable and reliable data. In addition to environmental data used for environmental models, in planning and licensing it can also be relevant to have economic data. Combining economic impacts like employment and income for the wider society, with economic data on environmental impact, like valuations, can make comparisons and trade‐off decisions easier and more transparent.^260^ Mikkelsen et al.^260^ showed the availability of relevant economic data on aquaculture. Economic data varies considerably between countries, but in general the data is poor, even for more developed countries such as those around the North Atlantic.

Earth observation (EO) is becoming increasingly important as a source of data to support monitoring of environmental conditions and planning and management of aquaculture at different spatial scales and resolutions.^261^ EO can provide spatial maps and time‐series of parameters such as temperature, chlorophyll, and total suspended solids, with considerable scope for use among planning and licensing frameworks to identify potential aquaculture zones and new sites.^262^, ^263^, ^264^, ^265^ For extractive species (e.g., mussels and oysters) that are reliant on the natural environment for food, EO is particularly useful for site scoping to provide a comprehensive overview of heterogeneous conditions within a bay or along a coastline. Thus, EO data can provide useful inputs to growth models to evaluate the spatial variation in potential sites,^262^ or combined with other factors within GIS‐based models for aquaculture planning and management,^114^ as shown by Barillé et al.^117^ However, for aquaculture and coastal management more broadly, there are still challenges in using EO, such as cloud cover and atmospheric correction, as reviewed by McCarthy et al.^266^

In situ data collection is rapidly evolving, particularly using new technology such as real‐time sensors that monitor conditions (e.g., sea temperature and dissolved oxygen variability^267^) that can be reviewed via mobile phones and computer at any time, and many parts of the aquaculture sector are developing techniques useful for daily operations.^268^, ^269^, ^270^ In addition to allowing quick responses to real‐time situations, data collected via sensors can be stored and archived, creating large datasets with long time‐series, that may also be useful for other areas of research if shared. Such information can be useful for industry and planning authorities as it provides an overview of conditions that can determine suitability of locations for site development or expansion. Licences may have requirements for post‐consent monitoring to allow initial decisions to be modified if found to be unrepresentative. Research in Scotland has shown that aquaculture producers often collect more data and do more analysis than minimum statutory requirements.^29^ New initiatives for environmental monitoring such as use of eDNA metabarcoding^271^, ^272^ could provide a more cost‐effective approach for benthic sampling than more traditional methods. As there are moves towards increasing transparency in the aquaculture sector, data may be made available to the public, to allow sharing of information on sustainability of the sector.^273^ It is important to recognise that online data portals can take a considerable amount of time and resources to develop and maintain, and there are also differences in how often they are updated and how recent the data are; however, they will increasingly play a role in data collection and presentation, especially if companies recognise the potential of wider sharing of such data.

## 3 CASE STUDY: PLANNING FOR SUSTAINABLE MARINE AQUACULTURE IN THE UNITED KINGDOM

Section 2 highlighted many of the issues associated with planning and licensing of sustainable marine aquaculture, with examples from across the world. A major, and often overwhelming, challenge for marine aquaculture in the 21st Century is the need to navigate a multitude of planning and licensing regulatory requirements. While the scientific literature provides an important overview of many of the issues, it should be recognised that not all issues are formally covered there. It is important, therefore, to understand some of the specific challenges involved in revising regulations, developing new policies, or implementing different approaches. To this end, some examples from the UK are given here based on the knowledge and experiences of the authors.

The UK, more formally known as the United Kingdom of Great Britain and Northern Ireland, is a country made up of four constituent countries: England, Northern Ireland, Scotland, and Wales. Aquaculture statistics are often reported at the UK level, (e.g., by the Food and Agricultural Organisation of the United Nations^274^), but aquaculture is a devolved matter, and each country has its own regulations and policies. As a consequence, and with differences in the environmental conditions and their suitability for fish and shellfish farming, aquaculture production varies considerably between the UK countries (see Box 5 and Table 2).

BOX 5 **UK marine aquaculture production statistics**

Aquaculture production statistics for the UK are available via international databases,^274^, ^275^ and separate statistics for Scottish aquaculture are published.^276^, ^277^, ^278^, ^279^ Although statistics for aquaculture within the other UK administrations are not currently published,^280^ they are available from the competent authorities who collect the data. No data have been collected from the seaweed sector emerging within the UK, and statistics are confidential for species with limited numbers of producers (e.g., Scottish halibut, Northern Irish salmon), necessitating estimation. Recognising these limitations, production statistics have been collated (Table 2) to provide a representative picture of recent marine aquaculture across the four administrations. A diversity indicator also was tabulated to enable comparisons (Table 2).

UK marine aquaculture produces about 200,000 tonnes per annum, with a first‐sale value of about £1 billion per annum (Table 2).^281^ Its value to the UK is considered greater due to associated upstream and downstream supply chains, spending on staff salaries, social and community benefits in remote and rural areas, and security of seafood supply.^282^, ^283^

The species farmed vary between the UK administrations, with production differing by two orders of magnitude (Table 2):

- Scotland generates 95% of UK marine aquaculture production worth £1 billion per annum. Although eight species are reported, production is highly skewed with Atlantic salmon being dominant.
- Northern Ireland generates 2% of UK marine aquaculture production worth £9 million per annum. Production is more evenly spread with three main species (mussels, Pacific cupped oyster, Atlantic salmon); this is the only UK administration outside Scotland currently producing finfish from sea‐water net‐pen sites.^284^
- England also contributes 2% of UK marine aquaculture production, worth £9 million per annum, solely from bivalve mollusc species.
- Wales generates 1% of UK aquaculture production, worth £3 million per annum, almost exclusively from mussels.

Comparable data for UK sea fisheries,^285^ indicates that: sea fisheries produce approximately double the volume (416,000 tonnes per annum), but with a lower first sale value (£0.76 billion per annum), across a far wider range of 39 species/groups (Shannon Diversity Index = 2.9) than UK marine aquaculture.

**TABLE 2:** Average annual volumes (in tonnes) for UK marine aquaculture production for the four administrative regions

English name	Scientific name	Scotland		Northern Ireland		England		Wales		UK total	
		Tonnes	%	Tonnes	%	Tonnes	%	Tonnes	%	Tonnes	%
Atlantic salmon	*Salmo salar*	180,912	94%	500	14%					181,412	89%
Rainbow trout*	*Oncorhynchus mykiss*	3775	2%							3775	2%
Atlantic halibut	*Hippoglossus hippoglossus*	67	0%							67	0%
Sea mussels	*Mytilus* spp.	7040	4%	2180	60%	2034	41%	2269	99%	13,523	7%
Pacific cupped oyster*	*Magallana gigas*	322	0%	926	26%	972	20%	18	1%	2239	1%
European flat oyster	*Ostrea edulis*	12	0%			11	0%			22	0%
Queen scallop	*Aequipecten opercularis*	4	0%							4	0%
Great Atlantic scallop	*Pecten maximus*	4	0%							4	0%
Japanese carpet shell (=Manila clam)*	*Ruditapes philippinarum*					12	0%			12	0%
Common edible cockle	*Common edible cockle*					1933	39%			1933	1%
Northern quahog (=Hard clam)*	*Mercenaria mercenaria*					1	0%			1	0%
Total tonnes p.a.		192,135	100%	3606	100%	4963	100%	2287	100%	202,991	100%
% Finfish		96%		14%		0%		0%		91%	
% Bivalve molluscs		4%		86%		100%		100%		9%	
% UK		95%		2%		2%		1%		100%	
Number of species		8		3		6		2		11	
Shannon Diversity Index (*H*′)		0.27		0.93		1.08		0.05		0.45	
Estimated value of production (p.a.)		£1,060,156,390		£9,079,308		£9,141,109		£2,875,305		£1,081,252,112	

*Note*: Figures represent averages for the five most recent years available (2016–2020) to smooth annual fluctuations. Non‐native species to UK indicated by *. Shannon Diversity Index (*H*′) calculated, with higher values indicating a more diverse and even spread.^286^ Corresponding £ value figures also included, corrected (inflation adjusted) to 2020 real prices. On‐shore production in tank systems of cleaner fish (lumpfish *Cyclopterus lumpus* and wrasse species *Labridae*) and crustaceans (European lobster *Homarus gammarus* and whiteleg shrimp *Penaeus vannamei*) is excluded.

**FIGURE 1:**
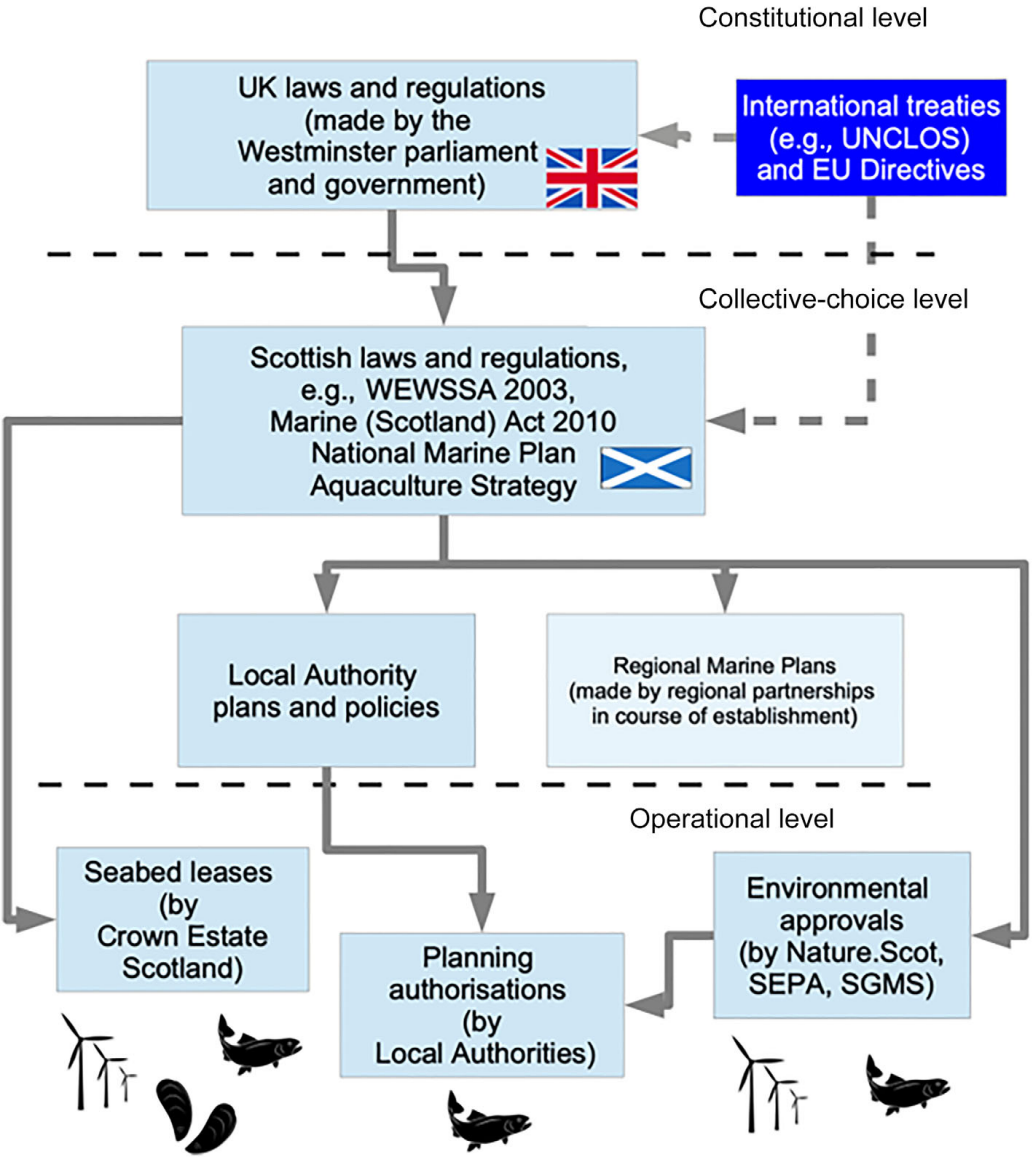
Some of the institutions (here referring to norms, policies and laws) and organisations (here referring to bodies that embody and implement the institutions) of polycentric governance relevant to planning and licensing for finfish and shellfish aquaculture and offshore wind‐farming in Scotland as part of the United Kingdom. At the *operational level*, only salmonid farming needs the full range of permissions. SGMS, Scottish Government's Marine Scotland directorate; UNCLOS, UN Convention on the Law of the Sea; WEWSSA, Water Environment and Water Services (Scotland) Act.

The UK demonstrates how complicated marine aquaculture development can be in multi‐layered governance structures. UK aquaculture is an example of polycentric governance, which refers to control by multiple institutions arranged in hierarchies. Figure 1 illustrates polycentric governance for different types of aquaculture in Scotland, including wind‐farming as an example of multiuse of space (though no commercial‐scale co‐location ventures exist in Scotland at present). This example is explained in more detail in the Data S1. In brief, within Scotland's inshore waters, operational governance, which lies at the bottom of the governance hierarchy, requires compliance with the regulations made by the Scottish Government under the Water Environment and Water Services (Scotland) Act (WEWSSA) of 2003 and implemented by The Scottish Environment Protection Agency (SEPA), and marine planning, overseen by Scottish Government's Marine Scotland Directorate (SGMS) under the Marine (Scotland) Act of 2010. The actors include local stakeholders and representatives of SEPA, SGMS, and so on. Local opinion about fish farming^77^ and multiuse^287^ is part of the context of licensing decisions. In the case of fish farming, such licensing is not, as might seem logical, managed as part of MSP, but is instead run by a county‐scale Local Authority as part of Town and Country Planning. The collective‐choice level is where society decides collectively on its options, in this case, relevant policies from the Scottish Government concerning fish farming and MSP, with authority provided by laws and regulations. The constitutional level of governance provides the rules and broad policy directions that influence the collective‐choice level. In the case of Scotland, this level includes the UK government and Supreme Court, and international agreements entered into by the UK. Before Brexit, the most important of these was the UK's membership in the European Union, which steered not only UK, but also Scots law. Scotland's WEWSSA explicitly implemented the European Water Framework Directive (WFD: 2000/60/EC), and its Marine (Scotland) Act implemented the European Marine Strategy Framework Directive (MSFD: 2008/56/EC). These provisions continue in Scots law, albeit subject to some uncertainty about changes in UK law.^288^ After Brexit, the UK remains a signatory to international conventions such as OSPAR, which helps protect the environments in the North East Atlantic,^289^ and the UN's Sustainable Development Goals (SDGs), where SDG 14 calls for member states to ‘conserve and sustainably use the oceans, sea and marine resources for sustainable development’.

The governance structure in Scotland shows how difficult it can be for individuals and organisations to be fully acquainted and compliant with all regulatory requirements. This difficulty is further complicated across the United Kingdom since there are different approaches in each of the countries and further variations depending on the location, species, and farming system used. Multiple licences from multiple organisations are required, and there is no one‐stop shop for planning and licensing in any of the countries where all permits, licences and permissions can be acquired. The challenges of navigating a complex licensing system are exacerbated by difficulties in finding information and guidance on the aquaculture planning and licensing process. The Griggs report,^12^ a review of aquaculture regulation in Scotland, noted problems trying to locate information on how to establish a fish farm in Scotland, recommending creation of a single source for guidance. Interestingly, this sort of initiative is found to some extent in England and in Wales through a series of regularly updated guidance documents that provide information on the regulatory requirements and licences required to establish an aquaculture site.^290^, ^291^ On the other hand, it is relatively easy to access information on the locations and licence conditions of all Scottish fish and shellfish farms via online portals,^292^ but there are no publicly accessible single‐source repositories for this information in the other countries, although one is planned for England.

The absence of publicly available data is not necessarily due to an unwillingness to share data. Data are often owned by different organisations (e.g., at least five organisations provide data for Scotland's Aquaculture website,^292^) and in some cases there may be terms and conditions that make data sharing difficult or illegal (e.g., in breach of data protection regulations). Given the challenges involved, co‐operation amongst the different organisations may be a way of facilitating access to data. This was the approach used for Scotland's Aquaculture website, which was developed in partnership with the different organisations involved in storing data relevant for planning and regulatory purposes.^292^ In each UK jurisdiction, a single one‐stop shop where all guidance and relevant data is found would be a useful step forward. It would also improve transparency within the sector and could help with social acceptance.

Any regulatory change takes time to develop and implement, and that transition phase creates an uncertain environment, which is a challenge for development. In Scotland, there has been a high level of uncertainty over the last 6 years as the aquaculture sector has undergone two government inquiries^293^, ^294^ and a series of reviews.^13^, ^295^, ^296^ Since the initial analysis of literature was completed for this present study, a major review of the Scottish aquaculture regulatory process has been published,^12^ that will lead to significant changes in planning and licensing. Crown Estate Scotland, who owns the seabed on behalf of the Crown and who leases it for development, also have announced major reform of rent and lease terms.^297^ While regulatory reviews are important because regulation should evolve over time, for a dynamic sector like aquaculture, the resulting stasis in decision‐making can radically impact progress. Frequent reviews also can erode trust in the system by all parties. Furthermore, for people outside the immediate policy/regulatory sphere, it is challenging to know what the most up‐to‐date policies and regulations are, which can lead to confusion and misunderstanding, particularly, but not exclusively, for new entrants. Frequent reforms and reviews increase the need for one‐stop‐shop information hubs.

Although aquaculture legislation is devolved, some UK‐level decisions will have direct and indirect effects on the sector. An example of this is Brexit. As the UK has left the European Union (EU), many laws and regulations are being reviewed, and there also are changes in economic policy and repositioning of priorities. A good illustration of this is the shellfish sector. Shellfish are grown in areas classified for food safety as either A, B or C, and the level of classification determines what treatment is required before the shellfish can be considered safe for human consumption. Many shellfish waters in the UK are Grade B, and when the UK was a member of the EU, shellfish from Grade B could be exported to the EU and depuration could take place in the EU.^298^ However, following Brexit, the EU now requires depuration before bivalves can be imported, or the bivalves must be from Grade A waters.^299^ This has led to a sudden inability to sell product for many producers, and significant drop in sales. Further, transition to the ‘new normal’, in which broader marine governance requirements, including planning and licensing requirements for aquaculture, are in place, will take considerable time to enact.^21^ For Northern Ireland, which shares a land border with the Republic of Ireland, Brexit could have consequences for marine spatial planning, particularly in the two transboundary embayments, Lough Foyle and Carlingford Lough,^300^ both large shellfish growing areas.

One of the key challenges is the dynamic nature of the marine environment and the need to understand how aquaculture interacts with the surrounding area. Each UK country has had an aquaculture industry for at least several decades, and the knowledge base has built up over the years and continues to evolve as new information is generated. However, strict regulatory systems and inflexible processes also create barriers to improved sustainability and resilience of the sector. Likewise, companies may invest in innovative technologies or develop new farming approaches that optimise use of the space and resources, but the regulatory framework may limit implementation. For example, an English shellfish producer has argued that legislation is too prescriptive and not based on the realities that the sector faces, which leads to lost opportunities and has left companies unable to respond to sudden changes.^16^ If evidence is available that can support a decision or action, then the knowledge should be translated into evidence‐based decision‐making by planners and regulators. However, this is not always the case, and reluctance to act can be a limitation to development. Brown et al.^301^ noted that a precautionary approach is used towards new sites within Marine Protected Areas (MPAs) in England, even though most shellfish farms have been operating without untoward impacts within English MPAs for many years.

## 4 RECOMMENDATIONS AND CONCLUSIONS

The wider literature review and the UK case study revealed many aspects of planning and licensing that need attention. The examples in the Boxes 1, 3 illustrate that issues are not always static, and decisions can have cascading and transformative effect on development of marine aquaculture at local, national, and international scales. Some of the challenges in aquaculture planning and licensing are internal to the sector, while other issues are due to external factors, which are often unexpected and difficult to predict. Nevertheless, common themes emerged from the literature, and these can be used to identify actions for the aquaculture community, policymakers and regulators (Table 3). There are overlaps in the recommendations as some actions can address more than one issue, but there will also be a need to consider details and nuances that are often behind the challenges faced by the sector.

**TABLE 3:** Recommendations for improvements in planning and licensing under each theme investigated in the review

Theme	Recommendations
Complicated and fragmented approaches to planning and licensing	A) Revision of national (or regional if mechanism available) licensing systems for consistency in requirements, implementation and licence term, and inclusion of social and technological considerations
	B) Central information about licensing and statutory rights available
	C) Coordination between agencies
	D) ‘One stop shop’
Property rights and licence to operate	A) Revision of national (or regional if mechanism available) licensing systems for consistency in requirements, implementation and licence term, and inclusion of social and technological considerations
	B) Central information about licensing and statutory rights available
	E) Consistent application of licence compliance, nationally and regionally.
	F) Stakeholder involvement in local, national and regional licensing review and processes
Competition for space and Marine Spatial Planning (MSP)	A) Revision of national (or regional if mechanism available) licensing systems for consistency in requirements, implementation and licence term, and inclusion of social and technological considerations (in relation to AZAs)
	F) Stakeholder involvement in local, national and regional licensing review and processes
	G) Development of allocated zones for aquaculture (AZAs)
	H) Implementation of environmental models and marine spatial planning tools for selection and development of aquaculture zones.
Emerging species and diversifying marine aquaculture production	
Seaweed farming	I) Economic and environmental feasibility studies in national and regional context (for seaweed)
	J) Research on efficiency and practicality of carbon capture
	K) Review of integration of provision of ecosystem services into licensing considerations, e.g., ‘green licensing’
Integrated Multi‐Trophic Aquaculture (IMTA)	A) Revision of national (or regional if mechanism available) licensing systems for consistency in requirements, implementation and licence term, and inclusion of social and technological considerations
	I) Economic and environmental feasibility studies in national and regional context (for IMTA)
	K) Review of integration of provision of ecosystem services into licensing considerations, e.g., “green licensing’
Offsetting with aquaculture	A) Revision of national (or regional if mechanism available) licensing systems for consistency in requirements, implementation and licence term, and inclusion of social and technological considerations
	I) Economic and environmental feasibility studies in national and regional context (for offsetting)
	K) Review of integration of provision of ecosystem services into licensing considerations, e.g., ‘green licensing’
Offshore aquaculture	A) Revision of national (or regional if mechanism available) licensing systems for consistency in requirements, implementation and licence term, and inclusion of social and technological considerations
	I) Economic and environmental feasibility studies in national and regional context (for offshore)
	L) Research on implications for offshore aquaculture on environment and animal welfare
Co‐location and multiuse platforms	A) Revision of national (or regional if mechanism available) licensing systems for consistency in requirements, implementation and licence term, and inclusion of social and technological considerations
	I) Economic and environmental feasibility studies in national and regional context
The need to address knowledge gaps and use of decision support tools	A) Periodic (10 years?) revision of national (or regional if mechanism available) licensing systems for consistency in requirements, implementation and licence term, and to take into account inclusion of social and new technological considerations
	J) Licensing authority and stakeholder engagement with academic and industry development of new decision support tools, to ensure fitness for purpose when licensing under review.
	K) Review of data formats, types, and availability for implementation in the licensing process following review.

*Note*: There is some overlap in the recommendations, which can be grouped for ease of interpretation (labelled A–K), but these letters and the order presented in the table should not be considered an indication of priority or importance.

Robust planning and licensing frameworks are a prerequisite for a successful and sustainable marine aquaculture sector. Since aquaculture is a diverse and dynamic sector it can be difficult to design and implement effective regulations, especially for emerging technology and production methods. Yet, production targets and visions for the sector will only be realised if planning and licensing frameworks support sustainable development in the most appropriate locations. Across the entire marine aquaculture sector there is recognition that many aspects of planning and licensing need improvement globally. Some parts of the sector, such as coastal salmon aquaculture, have received more attention than others. While this is to be expected given the economic importance of that form of aquaculture, it is also important to ensure there are initiatives in place to collect or generate the data and information needed to ensure that planning and licensing are fit for the myriad of other species and systems. ‘Planning and licensing’ is not a single‐issue and involves a wide‐range of interacting interdisciplinary considerations, so frameworks need to be fluid, versatile and adaptive. Most people, whether producers, regulators or the public, will face challenges navigating these complexities. Many of the findings from the wider review are seen in the United Kingdom, and while there are nuances to an area or issue, overarching themes are often comparable regardless of jurisdiction. Sharing experiences as well as ways in which bottlenecks and challenges have been addressed is important to ensure sustainable marine aquaculture in the 21st century.

## AUTHOR CONTRIBUTIONS

**Lynne Falconer:** Conceptualization; writing – original draft; writing – review and editing. **Karl Cutajar:** Writing – original draft; writing – review and editing. **Amalia Krupandan:** Writing – original draft; writing – review and editing. **Elisa Capuzzo:** Writing – original draft; writing – review and editing. **Richard A. Corner:** Writing – original draft; writing – review and editing. **Tim Ellis:** Writing – original draft; writing – review and editing. **Keith Jeffery:** Writing – original draft; writing – review and editing. **Eirik Mikkelsen:** Writing – original draft; writing – review and editing. **Heather Moore:** Writing – original draft; writing – review and editing. **Francis X. O'Beirn:** Writing – original draft; writing – review and editing. **Pauline O'Donohoe:** Writing – original draft; writing – review and editing. **Neil M. Ruane:** Writing – original draft; writing – review and editing. **Robyn Shilland:** Writing – original draft; writing – review and editing. **Paul Tett:** Writing – original draft; writing – review and editing. **Trevor C. Telfer:** Writing – original draft; writing – review and editing.

## CONFLICT OF INTEREST

The authors have no conflicts of interest to disclose.

## Supporting information


**Data S1.** Supporting Information.

## Data Availability

Data sharing is not applicable to this article as no new data were created or analyzed in this study.
