# Supplementary material for: Planning and licensing for marine aquaculture
Source: Rev Aquac. 2023 Jan 11;15(4):1374–404. doi: 10.1111/raq.12783 (PMC10947445; doi:10.1111/raq.12783)
Supplement: Supplementary file 1 — Data S1. Supporting Information. [file RAQ-15-1374-s001.pdf]

Supplementary Information

A sociological perspective

Sociological theory can illuminate several aspects of planning and licensing for aquaculture. However, there is more than one 'theory of society', as is exemplified by different meanings of the term *governance* in relation to Marine Spatial Planning (MSP) (Stojanovic and Gee, 2020). The sociological perspective used here is adapted from that of the Institutional Analysis and Development Framework (IADF) of Ostrom and colleagues (Ostrom, 2005, Ostrom, 2007, Ostrom, 2010, McGinnis, 2011, McGinnis and Ostrom, 2014), which provides a means of characterising Social-Ecological Systems (SES). Sociological parts of the IADF include the fundamental ideas of *institutions* and *action situations*, and the secondary concepts of *governance* and access rights to marine space and ecosystem services (i.e. *goods*). Recent aquacultural scholarship has been especially interested in these rights, and the problem of 'governing aquacultural commons' (Partelow et al., 2022). Here, however, we give most attention to the concept of *polycentric governance*, because this gives insight into several issues raised in section 2 of the main manuscript. Governance is understood here as the (legitimate) power-steering (Habermas, 1987, Habermas, 1996) of society, and is further defined, along with other key terms, in Table S1. Polycentric governance refers to control by multiple institutions at several levels of a hierarchy of power. It is illustrated in Figure S1, which relates to the UK case study in section 3 of the main manuscript, and includes wind-farming, to allow discussion of multi-use of sea-space.

The analysis of *action situations* (AS) lies at the core of the IADF. As an example, an application for permission to instal a marine fish farm in Scotland triggers an action situation that involves communication amongst a variety of *actors* and takes place under the constraints of various laws, regulations, policies and procedures to result in a binary outcome: either permission is granted or it is withheld. The collective term for the constraints is *institutions*, sometimes embodied in *organisations* such as Local Authorities, the Scottish Environment Protection Agency (SEPA), or the Scottish Government's Marine Scotland (SGMS) directorate. Institutions exist in the 'informational world' of Popper (1980), i.e., the Social System (SS) component of a SES, whereas organisations, consisting of people and facilities, are seen as part of the Ecological System (ES) component.

At the bottom of the governance hierarchy is the *operational level*. Ostrom (2007) and McGinnis and Ostrom (2014) set out schemes for characterising the SS and ES contexts of operational AS, and these have been adapted to aquaculture and applied to oyster cultivation in Maine, USA, by Johnson et al. (2019). In the Scottish example in Figure S1, *operational governance* requires compliance with the regulations made by the Scottish Government under the Water Environment and Water Services (Scotland) Act (WEWSSA) of 2003 and implemented by SEPA, and marine planning, overseen by SGMS under the Marine (Scotland) Act of 2010. The *actors* include local stakeholders and representatives of SEPA, SGMS, etc. Local opinion about fish farming (Billing, 2018) and multi-use (Billing et al., 2022) is part of the context of an AS. In the case of fish farming, the AS is not, as might seem logical, managed as part of MSP, but is instead steered by a county-scale Local Authority as part of Town and Country Planning.

The institutions that govern operational AS originate from AS at a higher level, where policies and regulations are made. This level is named *'collective choice',* as it is that level at which society decides collectively on its options. We are familiar with the taking of such decisions in democratically elected parliaments, but other governance systems are available, and all societies need institutions that ensure the survival of their SS (and, ideally, their SES). In countries without strong central government, the institutions that control AS may be local norms that are perturbed by interventions from large-scale markets, e.g. Galappaththi and Berkes (2015). In the Scottish example, relevant policies are those of the Scottish Government (SG), concerning fish farming and MSP.[1] The WEWSSA and the Marine (Scotland) Act help provide the authority for these regulations and policies.

The *constitutional level* of governance provides the rules and broad policy directions that influence collective-choice AS. In the case of Scotland, this level includes the UK government and Supreme Court, and international agreements entered into by the UK. Before Brexit, the most important of these was the UK's membership of the European Union, which steered not only UK, but also Scots law. Scotland's WEWSSA explicitly implemented the European Water Framework Directive (WFD: 2000/60/EC), and its Marine (Scotland) Act implemented the European Marine Strategy Framework Directive (MSFD: 2008/56/EC), and these provisions continue in Scots law, albeit subject to some uncertainty about changes in UK law (Harrison, 2021). After Brexit, the UK remains a signatory to international conventions such as OSPAR, which helps protect the environments in the NE Atlantic (Skjærseth, 2006), and the UN's Sustainable Development Goals, where SDG 14 calls for member states to "conserve and sustainably use the oceans, sea and marine resources for sustainable development".

Polycentricity refers not only to hierarchy, but also to the multiple and overlapping domains of institutions and the organisations that embody them. This condition causes some of the licensing complexities that are seen as frustrating aquacultural developments in many countries (Galparsoro et al., 2020) and which have led to the calls cited in the main text for 'one-stop shops' that can issue unified permissions. The problem is even more challenging in the case of shared use of sea space for electricity generation from offshore wind energy (OWE) and finfish or shellfish aquaculture. The potential for such multi-use has been explored by Calado et al. (2019) and Depellegrin et al. (2019), but is rendered difficult by sector-specific planning and regulatory regimes as well as by by the way each sector has developed. Scotland provides an example. Farming of fish and shellfish has developed in sheltered inshore waters during the last half-century (Griggs, 2022), with fish-farming developments regulated by Local Authority planning decisions. There has been no systematic approach to designating 'Allocated Zones for Aquaculture' as recommended by the FAO (Aguilar-Manjarrez et al., 2017). In contrast, OWE is largely regulated by SGMS, with Crown Estates Scotland releasing designated zones for wind-farming.

Nevertheless, institutions can be crafted to overcome some of these difficulties. An agreement between Scottish LA and environmental organisations (Anon, 2010) has allowed

---

[1] Sumarised by the Scottish Parliament's Information Centre: aquaculture-and-the-fourth-national-planning-framework

pulling together as a team led by the Local Authority, improving the permitting process for fish farm developments. The Griggs report (Griggs, 2022) has made further suggestions for improvement of aquacultural regulation, including more adaptive governance (Greenhill et al., 2020) and transferring the permitting role of Local Authorities to SGMS, which might also aid multi-use.

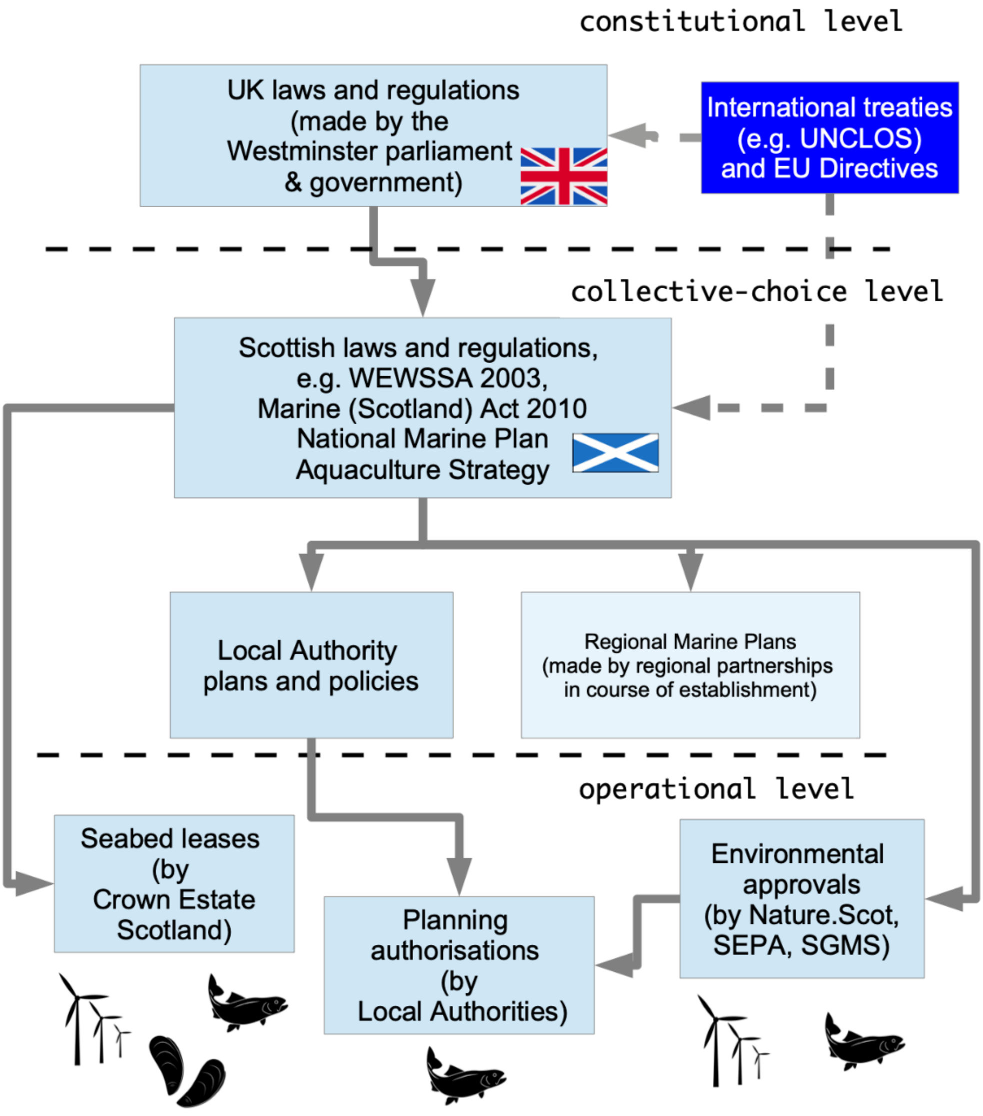

Figure S1: **Some of the institutions of polycentric governance** relevant to planning and licensing for fin-fish and shellfish aquaculture, and offshore wind-farming, in Scotland as part of the United Kingdom. Action Situations (AS) governed by these institutions occur at all levels. At the *operational level*, where relevant AS involve applications for leases, authorisations and approvals, only salmonid farming needs the full range of permissions. Acronyms: SGMS: Scottish Government's Marine Scotland directorate; UNCLOS: UN Convention on the Law of the Sea; WEWSSA: Water Environment and Water Services (Scotland) Act

Table S1: Key terms used in this section

**Social-ecological System (SES)**	An area in which human society interacts with 'nature', thus consisting of a **social system** within the 'informational world' (Popper, 1980) and an **ecological system** within the biophysical world ; see also Berkes et al. (1998) and Tett et al. (2013)
**Social system (SS)**	A communicative network (Luhmann, 1989) linking (human) persons and with rules that emerge from and shape personal actions (Sewell, 1992); Habermas (1987) subdivides SS into lifeworld and system, the latter steered by money (through *markets*) and power (through **governance**)
**Ecological system or Ecosystem (ES)**	Organisms (including humans as physical bodies) interacting (Lindeman, 1942) with their inanimate environment (including human constructions),
**Action Situation (AS)**	A finite communicative situation in which **actors** (acting for themselves or as agents of **organisations**) and governed by local and higher-order **institutions**, aim at, and often realise, actions in a SS, ES or SES that are intended to improve the functioning of these systems (adapted from McGinnis (2011), by adding purpose)
**Actor**	An embodied person communicating in society and having *agency* (the power of choice amongst possible actions and the ability to implement such choices in either or both SS or ES)
**Commons**	Any non-private **goods** and the open-access space containing them; the 'tragedy of the commons' (Hardin, 1968) refers to unsustainable use of these goods by self-interested actors: thus the commons needs governance (Partelow et al., 2022)
**Goods**	What the SS gets from the ES, classified (McGinnis, 2011) on basis of *subtractability* and *cost of exclusion;* common-pool goods are subtracted when used, and it is difficult to exclude users. Cf. Partelow et al. (2022), who included SS stuff (e.g. knowledge) in goods.
**Governance**	How power is directed to empower or constrain human action (Stojanovic and Gee, 2020); the governance system includes **institutions** and **organisations** (McGinnis and Ostrom, 2014)
**Institution**	A set of formal or informal norms, rules, laws, or procedures within a SS that constrain and empower **actors** and **organisations**
**Organisation**	An embodied **institution**, able to act in social and biophysical worlds

AGUILAR-MANJARREZ, J., SOTO, D. & BRUMMET, R. 2017. Aquaculture zoning, site selection and area management under the ecosystem approach to aquaculture: full document. Rome and Washington DC: FAO and World Bank Group.

BERKES, F., FOLKE, C. & COLDING, J. 1998. *Linking social and ecological systems. Management practices and social mechanisms for building resilience.,* Cambridge, Cambridge University Press.

BILLING, S.-L. 2018. Using public comments to gauge social licence to operate for finfish aquaculture: Lessons from Scotland. *Ocean & Coastal Management,* 165**,** 401-415.

BILLING, S.-L., CHARALAMBIDES, G., TETT, P., GIORDANO, M., RUZZO, C., ARENA, F., SANTORO, A., LAGASCO, F., BRIZZI, G. & COLLU, M. 2022. Combining wind power and farmed fish: Coastal community perceptions of multi-use offshore renewable energy installations in Europe. *Energy Research & Social Science,* 85**,** 102421.

CALADO, H., PAPAIOANNOU, E. A., CAÑA-VARONA, M., ONYANGO, V., ZAUCHA, J., PRZEDRZYMIRSKA, J., ROBERTS, T., SANGIULIANO, S. J. & VERGÍLIO, M. 2019. Multi-uses in the Eastern Atlantic: Building bridges in maritime space. *Ocean & Coastal Management,* 174**,** 131-143.

DEPELLEGRIN, D., VENIER, C., KYRIAZI, Z., VASSILOPOULOU, V., CASTELLANI, C., RAMIERI, E., BOCCI, M., FERNANDEZ, J. & BARBANTI, A. 2019. Exploring Multi-Use potentials in the Euro-Mediterranean sea space. *Science of The Total Environment,* 653**,** 612-629.

GALAPPATHTHI, E. K. & BERKES, F. 2015. Drama of the commons in the small-scale shrimp aquaculture in northwestern, Sri Lanka. *International Journal of the Commons,* 9(1)**,** 347-368.

GALPARSORO, I., MURILLAS, A., PINARBASI, K., SEQUEIRA, A. M. M., STELZENMÜLLER, V., BORJA, Á., O´HAGAN, A. M., BOYD, A., BRICKER, S., GARMENDIA, J. M., GIMPEL, A., GANGNERY, A., BILLING, S.-L., BERGH, Ø., STRAND, Ø., HIU, L., FRAGOSO, B., ICELY, J., REN, J., PAPAGEORGIOU, N., GRANT, J., BRIGOLIN, D., PASTRES, R. & TETT, P. 2020. Global stakeholder vision for ecosystem-based marine aquaculture expansion from coastal to offshore areas. *Reviews in Aquaculture,* 12**,** 2061-2079.

GREENHILL, L., STOJANOVIC, T. A. & TETT, P. 2020. Does marine planning enable progress towards adaptive governance in marine systems? Lessons from Scotland's regional marine planning process. *Maritime Studies,* 19**,** 299-315.

GRIGGS, R. 2022. A review of the aquaculture regulatory process in Scotland. Edinburgh: Scottish Government.

HABERMAS, J. 1987. *The Theory of Communicative Action. Volume 2: Lifeworld and System: A Critique of Fundamentalist Reason (translated by Thomas McCarthy). ,* Boston, MA/Cambridge, England, Beacon Press/Polity Press.

HABERMAS, J. 1996. *Between Facts and Norms: Contributions to a Discourse Theory of Law and Democracy. Translated by William Rehg.,* Cambridge, Mass., The MIT Press.

HARDIN, G. 1968. The Tragedy of the Commons. *Science,* 162**,** 1243-1248.

JOHNSON, T. R., BEARD, K., BRADY, D. C., BYRON, C. J., CLEAVER, C., DUFFY, K., KEENEY, N., KIMBLE, M., MILLER, M., MOEYKENS, S., TEISL, M., VAN WALSUM, G. P. & YUAN, J. 2019. A Social-Ecological System Framework for Marine Aquaculture Research. *Sustainability,* 11**,** 2522.

LINDEMAN, R. L. 1942. The Trophic-Dynamic Aspect of Ecology. *Ecology,* 23**,** 399-417.

LUHMANN, N. 1989. *Ecological communication (translated by Bednarz, John).* Cambridge, Chicago, Polity Press/University of Chicago Press.

MCGINNIS, M. D. 2011. An Introduction to IAD and the Language of the Ostrom Workshop: A Simple Guide to a Complex Framework. *Policy Studies Journal,* 39**,** 169-183.
MCGINNIS, M. D. & OSTROM, E. 2014. Social-ecological system framework: initial changes and continuing challenges. *Ecology and Society,* 19.
OSTROM, E. 2005. Understanding institutional diversity. *Understanding Institutional Diversity.* Princeton university press.
OSTROM, E. 2007. A diagnostic approach for going beyond panaceas. *Proceedings of the National Academy of Sciences,* 104**,** 15181-15187.
OSTROM, E. 2010. Beyond Markets and States: Polycentric Governance of Complex Economic Systems. *American Economic Review,* 100**,** 641-72.
PARTELOW, S., SCHLÜTER, A., O. MANLOSA, A., NAGEL, B. & OCTA PARAMITA, A. 2022. Governing aquaculture commons. *Reviews in Aquaculture,* 14**,** 729-750.
POPPER, K. 1980. Three worlds. The Tanner Lecture on Human Values, delivered at the University of Michigan, 7 April 1978. *In:* MCMURRIN, S. M. (ed.) *The Tanner Lectures on Human Values, volume 1.* Salt Lake City: University of Utah Press.
SEWELL, W. H. 1992. A Theory of Structure: Duality, Agency, and Transformation. *American Journal of Sociology,* 98**,** 1-29.
SKJÆRSETH, J. B. 2006. Protecting the North-East Atlantic: enhancing synergies by institutional interplay. *Marine Policy,* 30**,** 157-166.
STOJANOVIC, T. & GEE, K. 2020. Governance as a framework to theorise and evaluate marine planning. *Marine Policy,* 120**,** 104115.
TETT, P., SANDBERG, A., METTE, A., BAILLY, D., ESTRADA, M., HOPKINS, T. S., D'ALCALÀ, M. R. & MCFADDEN, L. 2013. Perspectives of Social and Ecological Systems. *Global Challenges in Integrated Coastal Zone Management.*
